# Residue 301-dependent epitope mapping reveals the molecular basis for GPV/MDPV serotype discrimination by neutralizing monoclonal antibody D1

**DOI:** 10.1186/s13567-025-01625-6

**Published:** 2025-10-16

**Authors:** Shifeng Xiao, Xiaoli Zhu, Min Zheng, Dangdang Jiang, Chaosong Zheng, Xiaoxia Cheng, Shao Wang, Guangju You, Shaoying Chen, Shilong Chen

**Affiliations:** 1 Institute of Animal Husbandry and Veterinary Medicine, Fujian Academy of Agricultural Sciences, No.247 Wusi Road, Fuzhou, 350003 China; 2 Fujian Animal Diseases Control Technology Development Center, Fuzhou, 350003 China; 3 College of Life Sciences, Fujian Agriculture and Forestry University, Fuzhou, 350002 China

**Keywords:** Waterfowl parvoviruses, neutralizing antibody, conformational epitope, AlphaFold 3, molecular dynamics

## Abstract

Waterfowl parvoviruses (goose parvovirus, GPV; Muscovy duck parvovirus, MDPV; Muscovy duck-origin goose parvovirus, MDGPV; short beak and dwarfism syndrome virus, SBDSV) are significant pathogens in waterfowl. The monoclonal antibody D1 specifically recognizes GPV-serotype parvoviruses (including GPV, SBDSV, and MDGPV) but cannot bind MDPV. Thus, Western blot and indirect immunofluorescence assays confirmed that D1 recognizes a conformational epitope, and residue 301 is critical for its discriminatory binding. Subsequent integrative approaches—combining AlphaFold 3-predicted structural modeling, molecular dynamics simulations, immunofluorescence, and transmission electron microscopy—revealed that VP3 residues 65 and 296 also contribute to the D1 epitope. Position 301 is a conserved divergent site (Asn in GPV, MDGPV, and SBDSV versus Lys in MDPV). Therefore, steric hindrance from lysine (K301) likely explains the inability of D1 to recognize MDPV. These findings elucidate the D1–VP3 binding mode, advancing our understanding of immune recognition mechanisms in waterfowl parvoviruses.

**Supplementary Information:**

The online version contains supplementary material available at 10.1186/s13567-025-01625-6.

## Introduction

Goose parvovirus (GPV), known as Derzsy’s disease virus [1], causes intestinal obstruction in gosling but rarely infects Muscovy ducklings despite circulating in China since 1956 [2]. Goose parvovirus belongs to the *Anseriform dependoparvovirus 1* [3], has a single-strand DNA genome (~5.1 kb) that consists of inverted terminal repeat sequences (ITRs) at both ends, regulator protein sequences (Rep1-2), and 19 bp undefined linker and viral capsid sequences (VP1-3), ranging from 5′ to 3′, respectively. The VP proteins and Rep are derived from the same gene that ends via a common termination codon. However, VP3 is a cleavage product and a major component of the capsid protein, accounting for >70% of capsid proteins [4–6]. Individually expressing each VP protein can result in assembly into virus-like particles (VLPs).

Besides GPV, there are three other main types of waterfowl parvoviruses, namely Muscovy duck parvovirus (MDPV) [5], short beak and dwarfism syndrome virus (SBDSV) [7–13], and Muscovy duck-origin goose parvovirus (MDGPV) [14, 15]. These parvoviruses have different host susceptibilities, clinical manifestations, genes, and serotypes. Muscovy duck parvovirus has been among the most important pathogens of Chinese Muscovy ducks since 1985 [5], infecting ducklings < 3 weeks of age, inducing diarrhea with intestinal mucosal bleeding and pancreatic white necrotic spots [5, 8, 16]. Further, SBDSV, a new branch of GPV, infects most waterfowl, causing weight loss [17] and induces short beak and dwarfism syndrome in mule and Cherry Valley ducklings [8–12]. Additionally, MDGPV, a recombinant virus of GPV and MDPV, exhibits a similar serology to GPV [18–21] and can infect Muscovy ducklings and gosling [19, 22]. The strains have 79.6–96.7% similarity of their VP genes, with GPV and MDPV having the largest difference (~80%), with a nonideal cross-protection effect [21, 23]. These slight VP gene differences have affected the prevention and treatment of the disease.

Thus, immunological diagnostic techniques based on monoclonal antibodies, such as enzyme-linked immunosorbent assay (ELISA) and colloidal gold strip tests, are important for disease diagnosis. A murine-derived monoclonal antibody, D1, previously developed in our laboratory [24], can specifically recognize GPV serology-type parvoviruses (GPV, SBDSV, and MDGPV) but not MDPV. D1 is an IgG1 subtype monoclonal antibody with a significantly higher neutralization titer (9 log_2_) to GPV than other reported waterfowl parvoviruses antibodies (e.g., 1:80 [25] and 7–8 log_2_ [26]). This high neutralization titer suggests that D1 targets a dominant antigenic epitope capable of eliciting a robust immune response in GPV-related parvoviruses. However, D1 recognition sites remain unknown.

Antibody recognition epitopes include linear and conformational epitopes. Linear epitope identification is achieved by continuously truncating the antigen peptide chain. However, conformational epitopes are more complicated, requiring protein crystallization, X-ray crystal diffraction, and cryo-electron microscopy. In this study, D1 recognized conformational epitopes of the VP3 protein of GPV serology-type parvoviruses. Further, this study obtained different VP3 amino acid mutants through the insect baculovirus expression system. The key epitopes were precisely confirmed by the D1–VP3 mutant binding using IF, and the epitopes were mutated into different amino acids to verify the factors that affect binding. AlphaFold 3 predicted the structures of the wild-type and mutant D1–VP3 complexes before subjection to the molecular dynamics (MD) study. Next, alanine scanning was performed in combination with the predicted interface, and the binding situation was verified by IF. Overall, this study reports the first conformational epitope analysis of waterfowl parvoviruses, which may be helpful in diagnosis and treatment. The results provide a low threshold and feasible way to study conformational epitopes.

## Materials and methods

### Antibodies, viruses, and cells

The monoclonal antibody D1 (IgG1 isotype), targeting the VP3 protein of GPV, was previously generated by immunizing BALB/c mice with purified GPV antigen, followed by hybridoma technology and screening via ELISA for GPV specific binding, as described in [24]. Murine hyperimmune serum against GPV and MDPV was prepared by immunizing BALB/c mice with a 1:1 mixture of purified GPV and MDPV antigens emulsified in Freund’s complete adjuvant. Mice were immunized three times at 2-week intervals. Two weeks after the third immunization, blood was collected from the tail vein, and serum was obtained by centrifugation. Immunofluorescence (IF) and western blotting (WB) were performed using FITC-conjugated goat anti-mouse IgG antibody (Biodragon, Suzhou, China) and HRP-conjugated goat anti-mouse IgG antibody (Bioss, Beijing, China), respectively. However, the GPV NP5 strain (GenBank accession no. PQ272760.1) and MDPV P1 strain (GenBank accession no. KU844282.1) were locally isolated in the laboratory. *Spodoptera frugiperda* Sf9 insect cells (Invitrogen, MA, USA) were cultured in a SIM SF expression medium (Sino Biological, Beijing, China) at 27 ℃.

### VP3 protein expression

The VP3 protein of GPV NP5, MDPV P1, and other mutants was prepared using the insect baculovirus expression system described before [27]. Specifically, viral DNA was extracted using the FastPure Viral DNA/RNA Mini Kit (Vazyme, Nanjing, China). The VP3 sequence of GPV NP5 and MDPV P1 were amplified using the HTB-NP5F/R and HTB-M3F/R primer pair (Additional file 1) before cloning onto the pFastBac HTB vector (cut by BamHⅠ and NotⅠ restriction enzyme, from Takara, Liaoning, China) using the Seamless cloning kit (Biosharp, Anhui, China). The recombinant plasmid was transformed into DH5α (Biomed, Beijing, China) and identified by sequencing (Sangon, Shanghai, China). The correctly sequenced plasmids were extracted and transformed to DH10Bac (Biomed, Beijing, China) to generate the recombinant bacmid, which was identified by blue–white spot screening and PCR using universal PUC/M13 primers. Next, the verified recombinant bacmids were transfected into Sf9 insect cells seeded at 8 × 10^5^ cells/well in six-well plates using SF9/SF21 Cell Culture Media SF-SFM (Womei, Suzhou, China) with LipoInsect™ transfection reagent (Beyotime, Shanghai, China) at 27 °C. Finally, recombinant baculoviruses were passaged to the third generation and used to infect Sf9 cells seeded at 5 × 10^4^ cells/well in 96-well plates at a multiplicity of infection (MOI) of 5 in SF9/SF21 Cell Culture Media SF-SFM. Infected cells were incubated at 27 °C for 96 h to express VP3 proteins, which were used directly for IF as described later.

### Identifying the D1-recognized epitope type

The D1 epitope type was identified by WB and IF. For the WB, total proteins were extracted from Sf9 cells infected with GPV-NP5-VP3 or MDPV-P1-VP3 recombinant baculoviruses, alongside control Sf9 cells and were resolved using the SDS-PAGE sample loading buffer (Beyotime, Shanghai, China). Next, 5% β-mercaptoethanol was added to the protein solution and boiled for 10 min to ensure complete protein denaturation. The 10% FuturePAGE™ Protein precast gels (ACE biotechnology, Jiangsu, China) were used for the SDS-PAGE, and samples were transferred onto NC membranes (Millipore) using the JY-ZY3 Semi-dry transfer blotting instrument (Junyi Electrophoresis). The D1 monoclonal antibody and HRP-conjugated goat anti-mouse IgG antibody were used as the primary and secondary antibodies, respectively. Further, the controls were the murine hyperimmune serum of GPV, MDPV, and HRP-conjugated goat anti-mouse IgG antibody.

For the IF, VP3 recombinant baculovirus-inoculated Sf9 cells and parvovirus-infected MDEFs (Muscovy duck embryo fibroblast cells) were fixed using cold acetone and incubated with D1 (diluted 1:150 in PBS), followed by FITC-conjugated goat anti-mouse IgG antibody (diluted 1:75 in PBS). The control was the murine hyperimmune serum of GPV (diluted 1:150 in PBS), while MDPV (diluted 1:150 in PBS) and FITC-conjugated goat anti-mouse IgG antibody (diluted 1:75 in PBS) were the primary and secondary antibodies, respectively. The nucleus was stained using DAPI (Beyotime, Shanghai, China), and plates were examined using an All-in-One Fluorescence Microscope BZ-X800 (KEYENCE, Osaka, Japan). Whenever the control is established, and the WB result on D1 is positive, D1 recognizes a linear epitope. Otherwise, it recognizes a conformational epitope.

### The VP3 amino acid sequence analysis

The VP3 amino acid sequences of D1-recognized GPV NP5, MDGPV PT, SBDSV M15, D1-unrecognized MDPV P, and MDPV P1 strains were aligned and analyzed by Jalview [28]. Thus, D1 has differential epitopes in the differential amino acid sites between D1-recognized and D1-unrecognized strains.

### Differential epitope screening

Mutants were expressed using the insect baculovirus expression system to determine the differential epitopes influencing D1 binding. The VP3 proteins (543 amino acids (aa)) of the D1-recognized GPV NP5 and MDPV P1 were the basic reference templates. First, the VP3 of GPV NP5 was set to three regions, viz. 1 (1–229 aa), 2 (230–341 aa), and 3 (342–543 aa), called G1, G2, and G3, respectively. The same applied for MDPV P1, M1, M2, and M3, respectively. These regions were freely combined to get recombined VP3 sequences: G1G2M3, G1M2M3, G1M2G3, M1G2M3, M1G2G3, and M1M2G3. These recombined VP3 sequences were expressed by the insect baculovirus expression system using pFastBac HTB plasmids, and the IF experiment was used to evaluate whether VP3 bound to the D1 antibody. Transmission electron microscopy (TEM) characterized the assembly of the recombined VP3 protein-VLPs in Sf9 cells [27] to verify whether the allelic amino acid substitution mutants affect virus assembly. Furthermore, the ^283^VRAYPGGTN^291^ of the NP5 VP3 was replaced by FETKEGDSS and proven unable to assemble into VLPs before being used to test whether the D1-recognized region is in the VP3 protein monomer or at the polymer junction. Then, different GPV NP5 and MDPV P1 sites were replaced one by one to identify the residues that specifically affect binding. The corresponding pFastBac HTB plasmids were constructed using the Mut Express II Fast Mutagenesis Kit V2 (Vazyme, Nanjing, China) and used for protein expression; their binding to D1 mab was tested using IF. Additional file 1 contains the relevant primers.

### Site-directed mutagenesis of related epitopes

Site-directed mutagenesis was performed in NP5 VP3 by substituting the relative epitopes with residues representing diverse physicochemical properties: glutamate (Glu/E, the side chain is –(CH_2_)_2_–COOH, negative charge, hydrophilic) and arginine (Arg/R, the side chain is –(CH_2_)_3_–NH–C(NH_2_)_2_^+^, positive charge, hydrophilic). The purpose was to test electrostatic complementarity to determine the mechanism by which related epitopes affect antigen–antibody binding. Tryptophan (Trp/W, the side chain is indole ring, neutral, hydrophobic), glycine (Gly/G, no side chain, neutral, cannot form hydrogen bonds), and alanine (Ala/A, the side chain is –CH_3_, neutral, hydrophobic) was used to evaluate spatial constraints. Next, glutamine (Gln/Q, side chain is –(CH_2_)_2_–CONH_2_, hydrophilic, can form hydrogen bonds), Gly, and Ala (cannot form hydrogen bonds) were used to determine whether hydrogen bonds were involved. Trp, Ala, Gln, and Arg mutations can also verify the influence of hydrophobicity and hydrophilicity on binding. These mutants were expressed through the insect baculovirus expression system using pFastBac HTB plasmids, besides testing D1 binding using IF. Additional file 1 contains the relevant primers.

### Structure prediction

Complex structure prediction was performed in AlphaFold 3 using the sequences of the NP5 VP3 and the D1 variable region [29]. The variable regions of the D1 heavy chain (VH) and light chain (VL) were sequenced using DetaiBio (Nanjing, China). Next, predictions were evaluated across 20 independent runs, and rank0 models were selected for further analysis. The predicted models were evaluated as: > 0.75 pTM + ipTM score; an antigen–antibody binding interface containing aa301 of VP3, where possible, CDR1, CDR2, and CDR3 of the D1 monoclonal antibody. Finally, the predicted structure of the GPV capsid-bound antibody was computationally aligned with the known GPV capsid structure (PDB ID: 9ME0) [30] to validate the structural compatibility of the complex. The model with the highest chain_pair_iptm score was chosen as the WT model of NP5-VP3-D1. The site-directed mutational models of N301A, N301E, N301R, N301G, N301Q, N301K, and N301W were constructed using PyMOL (vision 3.0.3) based on the WT model. Finally, molecular dynamics analysis was performed on the wild-type and site-directed mutation models.

### Molecular dynamics study

The molecular dynamics study was conducted using GROMACS (version 2020.6) at 25 °C (298 K) with the amber99sb-ildn force field to analyze the differences between wild-type antigen–antibody complexes and mutants [31]. The complex was immersed at the center of an SPCE water cube box, maintaining a minimum distance of 15 Å between the complex and all box edges. Next, the charges from the cation Na^+^ and anion CL^−^ neutralization system were added, and the energy of each system was separately minimized using the steepest descent minimization algorithm until the maximum force reached 100 kJ/mol/nm. The NVT (constant number of particles, volume, and temperature) equilibration for each system was run for 500 ps with a time step of 2 fs, followed by NPT (constant number of particles, pressure, and temperature) equilibration for 1000 ps. After the system was well equilibrated at the desired temperature, 298 K, and pressure 1 bar, three independent simulations of the finished product sampling were run for 300 ns under the same conditions. The parameters used to evaluate the antigen–antibody system were as follows:

- The root mean square deviation (RMSD) of the protein backbone atoms was calculated relative to the initial minimized structure to evaluate the structural stability.
- The root mean square fluctuation (RMSF) of the antigen residues was calculated to quantify local conformational flexibility.
- The buried surface area (BSA) at the antigen–antibody interface was calculated as BSA = (SASA_antigen_ + SASA_antibody_) − SASA_complex_, where SASA denotes the solvent-accessible surface area computed with a 1.4 Å probe radius (default).
- Hydrogen bond occupancies were quantified using Visual Molecular Dynamics (VMD, version 1.9.4a53) [32] over the equilibrated phase (50–100 ns, 5000 frames), with the criteria of donor–acceptor distance ≤ 3.5 Å and angle ≥ 150°.
- The total binding energy (Δ*G*_bind_) between the antigen (receptor) and antibody (ligand) was calculated using the MM/PBSA method [33], and key interfacial residues within a 4-Å cutoff distance were analyzed. Descriptions that were not directly defined by the MM/PBSA results are as follows:
  $${\text{Hydrophobic}}\;{\text{contribution}} = {\text{ESURF}}\left( {{\text{nonpolar}}\;{\text{solvation}}\;{\text{energy}}} \right)$$
  $${\text{Electrostatic}}\;{\text{contribution}} = {\text{EEL}}\left( {{\text{Electrostatic}}\;{\text{energy}}} \right) + {\text{EGB}}\left( {{\text{Electrostatic}}\;{\text{and}}\;{\text{polar}}\;{\text{solvation}}\;{\text{energy}}} \right)$$

### Validation of the relevant residues involved in binding

Systematic alanine scanning mutagenesis was performed, targeting residues that fulfilled either of the following criteria: (a) binding free energy contribution > 4 kcal/mol in any variant (wild-type or D1 antibody-binding mutants), (b) binding free energy contribution > 2 kcal/mol presence in ≥ 80% of analyzed variants, or (c) involvement in hydrogen-bond networks in ≥ 80% of the analyzed variants. The purpose was to rigorously validate the key binding residues in the mAb D1 binding process identified by molecular dynamics simulations. The mutant proteins were prepared as described above, and the D1 to mutant proteins combination was also tested by IF. The mutants with significantly altered recognition sites containing mAb D1 and hyperimmune serum were subsequently analyzed by SDS-PAGE to confirm correct protein expression and by TEM to assess VLP folding. Epitope-associated residues were evaluated for conservation through multiple sequence alignment of VP3 proteins from representative strains (MDPV, GPV, SBDSV, and MDGPV) by Jalview.

### Statistical analysis

Data are expressed as mean ± standard deviation (SD). One-way analysis of variance (ANOVA) was used to compare BSA among wild-type and mutant VP3 proteins, with post hoc Tukey’s test for multiple comparisons. Statistical analyses were performed using GraphPad Prism (version 8.0.1). Differences were considered highly significant at *p* < 0.01 and significant at 0.01 < *p* < 0.05.

## Results

### Monoclonal antibody D1 recognizes conformational epitope

**Figure 1:**
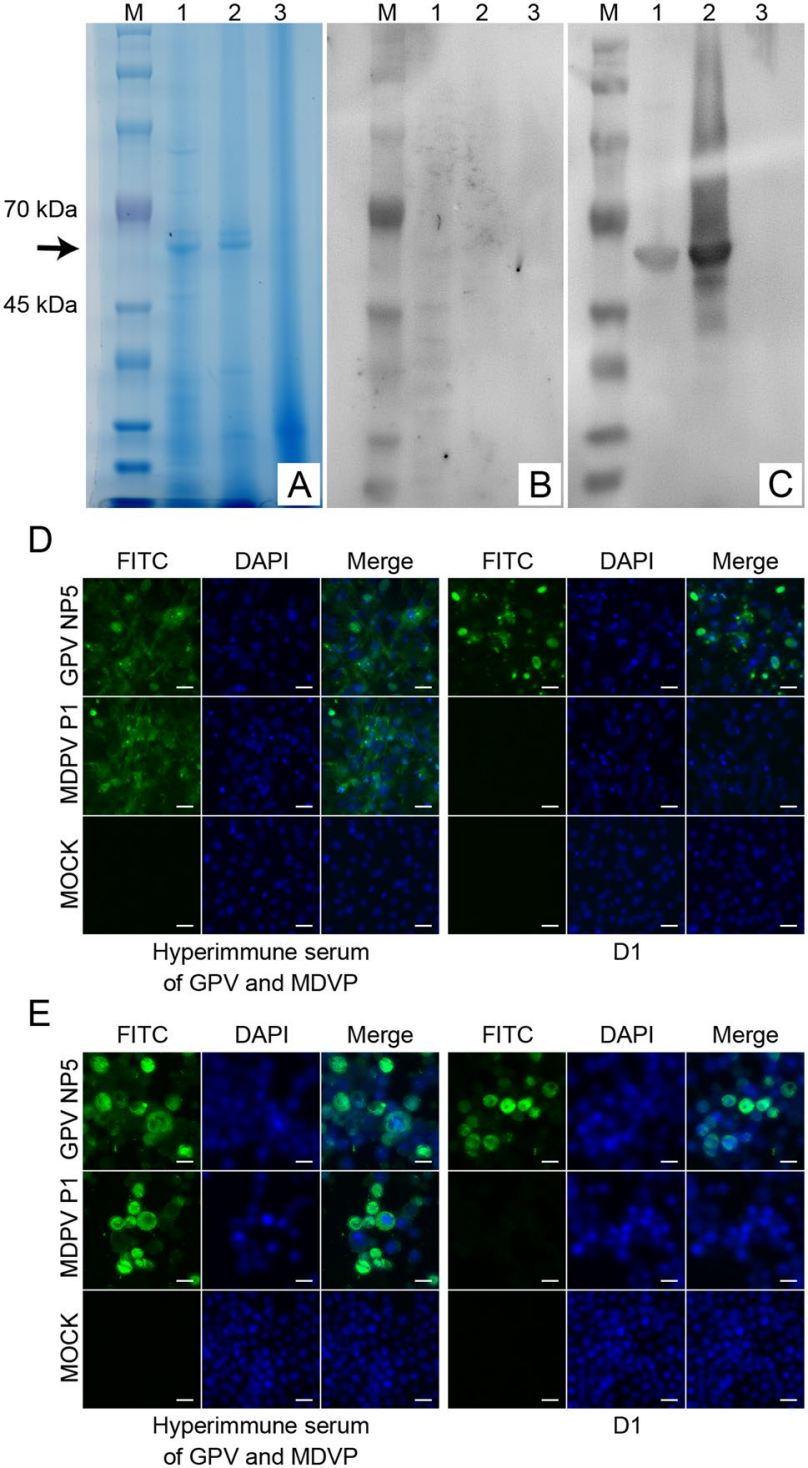
**Identification of mAb D1-recognized epitope types.** SDS-PAGE (**A**) and Western blotting (**B**, **C**) analysis of whole proteins of GPV (lane 1) and MDPV (lane 2) expressed by the baculovirus expression system, control Sf9 cells (lane 3), and protein Marker (M). The monoclonal antibody D1 (**B**) and mouse hyperimmune serum (**C**) were used as primary antibodies, and the HRP-conjugated goat anti-mouse IgG antibody was used as the secondary antibody. Indirect immunofluorescence detected mAb D1 recognition on MPDV P1 and GPV NP5 strains infected with MDEFs (**D**), P1 VP3-recombinant baculovirus (**E**), and NP5 VP3-recombinant baculovirus-infected Sf9 cells (**E**). Scale bars (white), 20 μm (**D**, **E**).

Western blot and IF experiments were used to determine whether the D1 mab recognizes linear or conformational epitopes. β-Mercaptoethanol was added to the DTT-containing protein loading buffer in SDS-PAGE and WB experiments to ensure the complete disruption of disulfide bonds, a process in VP3 protein denaturation. As shown by SDS-PAGE (Figure 1A), the size of the VP3 protein fused with the 6 × His tag was consistent with the theoretical size of 63.4 kDa. However, the D1 antibody failed to bind the denatured VP3 protein transferred to the NC membrane (Figure 1B), while the murine hyperimmune serum of GPV and MDPV bound normally (Figure 1C). The IF results showed positive results of D1 for both GPV NP5 infected MDEFs and NP5 VP3-recombinant baculovirus-infected Sf9 (Figures 1D, E). The IF experiment did not completely destroy the spatial structure of the protein by fixing the cells with methanol. Thus, the denaturing agent was added to destroy the spatial structure during SDS-PAGE and WB experiments, suggesting that D1 recognizes conformational rather than linear epitopes.

### Amino acid differences in VP3 of selected GPV, MDPV, MDGPV, and SBDSV strains for mAb D1 recognition

**Figure 2:**
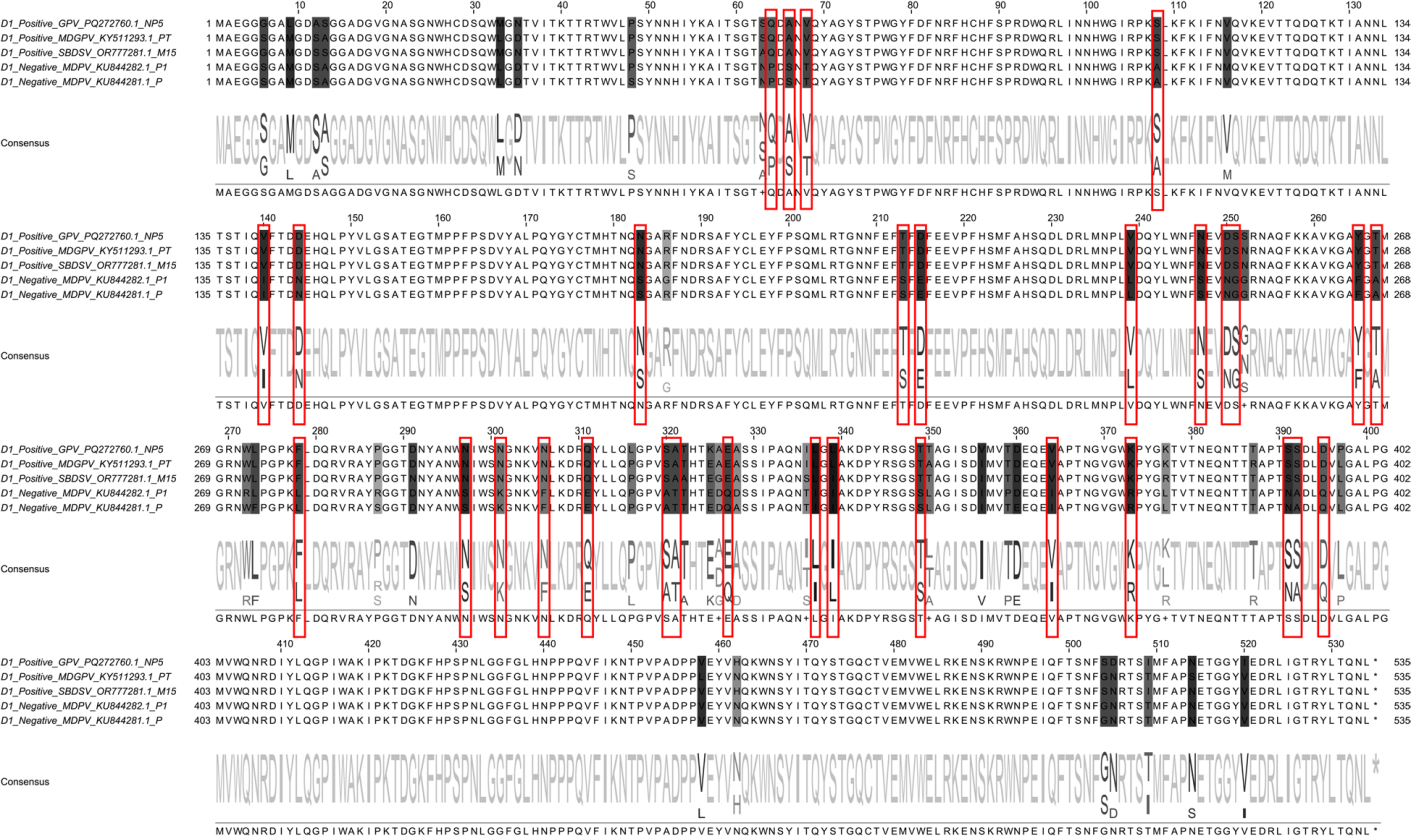
**Comparison of VP3 sequences from mAb D1-recognized and unrecognized strains.** Red boxes mark key differing residues.

The VP3 sequences of D1-recognized GPV NP5, MDGPV PT, and SBDSV M15, D1-unrecognized MDPV P, and MDPV P1 strains shared 66 different amino acid sites (Figure 2). However, D1 recognition and D1 non-recognition groups had only 31 different amino acids, the differential D1 epitopes.

### Role of VP3 aa301 in mAb D1 binding to GPV and MDPV

**Figure 3:**
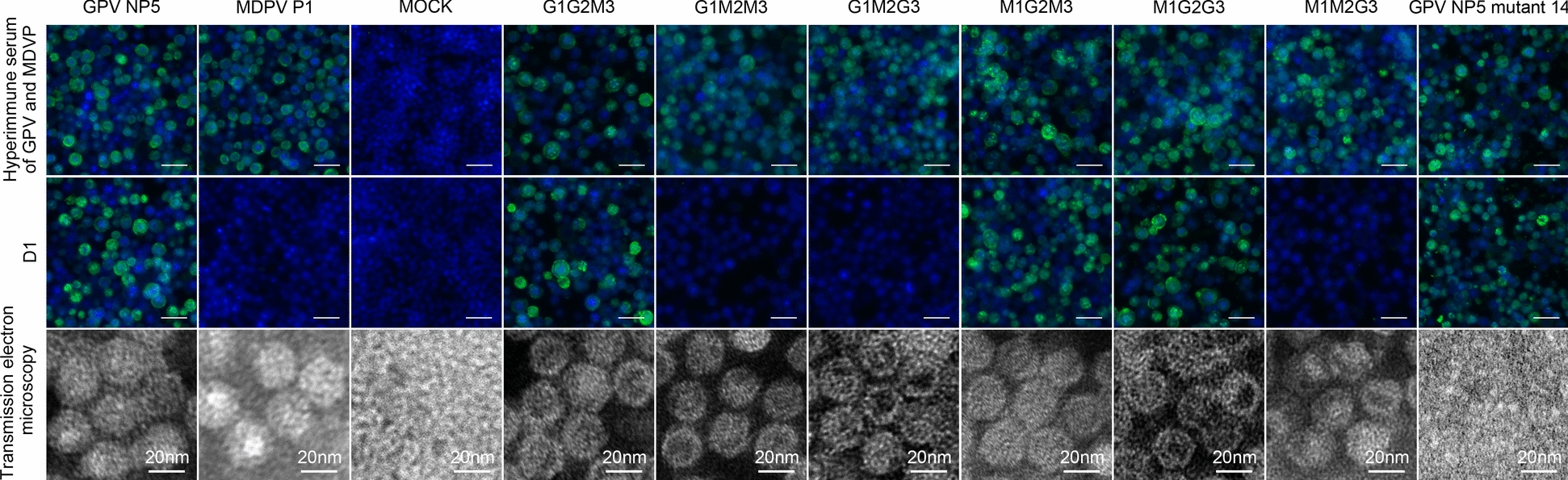
**Indirect immunofluorescence of the mAb D1 recognition on Sf9 cells expressing different VP3 protein types and the corresponding transmission electron microscope (TEM) results.** The VP3 of GPV NP5 was set to three regions, region 1 (1–229 aa), region 2 (230–341 aa), and region 3 (342–543 aa), referred to as G1, G2, and G3. The regions for MDPV P1 were M1, M2, and M3. These regions were freely combined to get recombined VP3 sequences: G1G2M3, G1M2M3, G1M2G3, M1G2M3, M1G2G3, and M1M2G3. The NP5 VP3 mutant 14 was derived from NP5 VP3 with the sequence VRAYPGGTN (aa 283–291) replaced by FETKEGDSS. The monoclonal antibody D1 and mouse hyperimmune serum and FITC conjugated Goat Anti-Mouse IgG antibody were used as primary and secondary antibodies, respectively. Scale bars, 50 μm for immunofluorescence images (unlabeled) and 20 nm for TEM images (labeled).

D1 binds to G1G2M3, M1G2M3, and M1G2G3 but not to G1M2M3, G1M2G3, and M1M2G3, suggesting that the main epitopes affecting D1 binding might be in region 2 (230–341 aa) of VP3 (Figure 3). Moreover, all six recombined VP3 contained self-assembled VLPs. Therefore, substituting the different sites had little effect on VLP formation. Most importantly, the NP5 VP3 mutant named 14, which self-assembled into VLPs, showed an IF positive result to D1 (Figure 3). Therefore, the D1-recognized region was in the monomer VP3 protein rather than the junction of the polymers.

**Figure 4:**
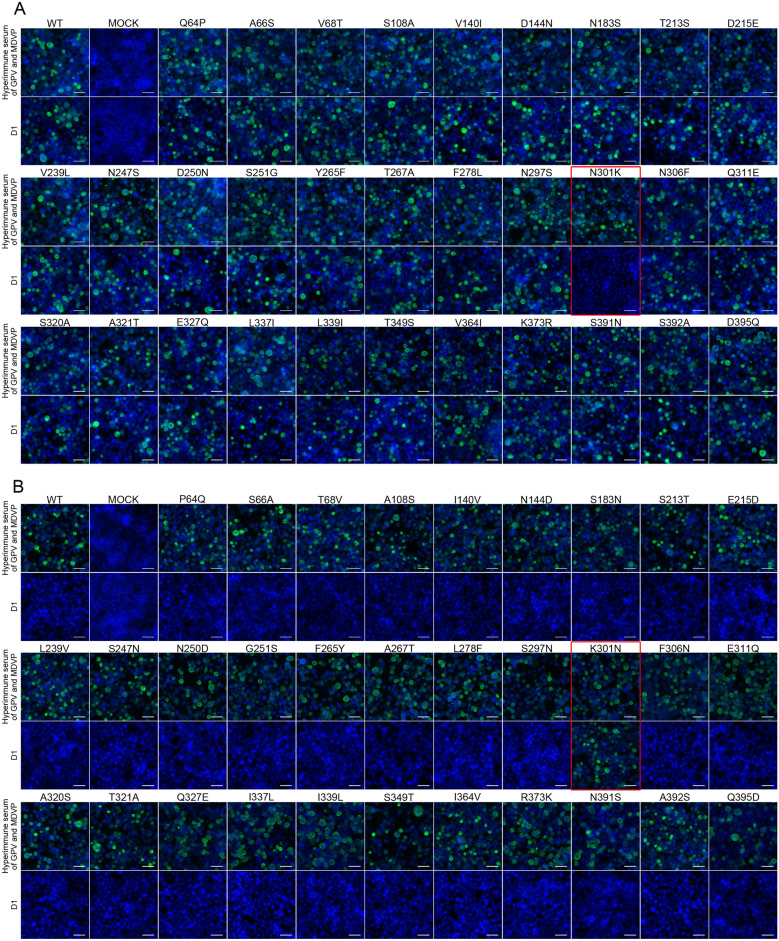
**Indirect immunofluorescence of monoclonal antibody D1 on differential site replacement VP3 binding.** (**A**) The amino acids at the relevant GPV NP5 sites were substituted with those of MDPV P1. (**B**) The amino acids at the relevant MDPV P1 sites were substituted with those of GPV NP5. Monoclonal antibody D1 and mouse hyperimmune serum were used as primary antibodies, while FITC-conjugated goat anti-mouse IgG antibody was used as the secondary antibody. Scale bars (white), 50 μm.

D1 bound to all single-site mutants GPV NP5 except N301K (Figure 4A) and could only bind to the K301N mutants of MDPV P1 (Figure 4B). Therefore, asparagine (Asn/N) and lysine (Lys/K) at position 301 in VP3 was important for mAb D1 binding.

### Site-directed mutagenesis of aa301 on VP3

**Figure 5:**
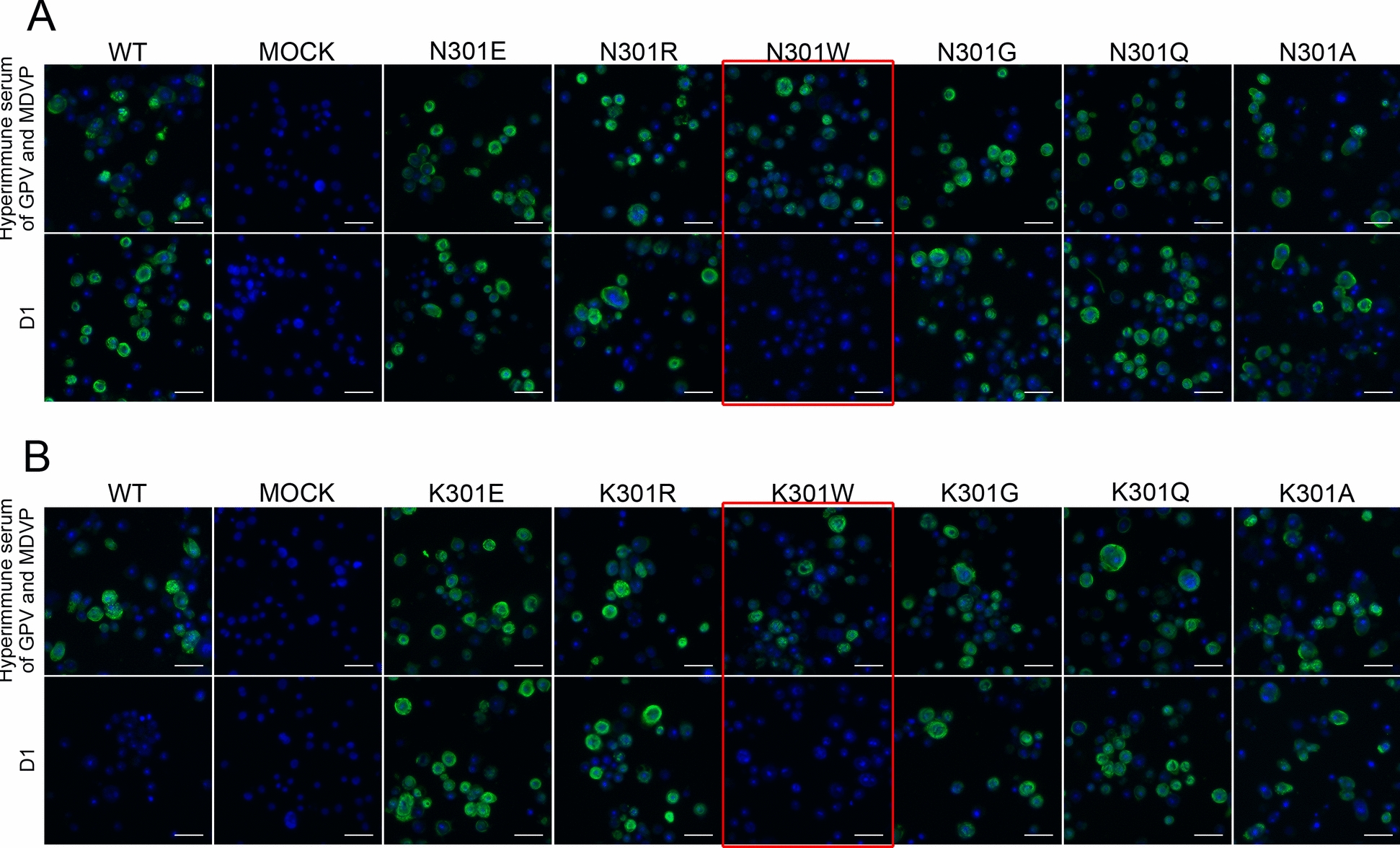
**Indirect immunofluorescence of monoclonal antibody D1 on the binding of different site-directed mutants of the aa301 of NP5 VP3.** (**A**) Site-directed mutants of the GPV NP5 strain. (**B**) Site-directed mutants of the MDPV P1 strain. Monoclonal antibody D1 and mouse hyperimmune serum were used as primary antibodies, and FITC-conjugated goat anti-mouse IgG antibody was used as the secondary antibody. Scale bars (white), 50 μm.

D1 bound to all the aa301 of NP5 and P mutations except N301W NP5 and K301W MDPV (Figure 5). Moreover, D1 monoclonal antibody recognition of VP3 was dependent on residue 301 identity: Asn, Ala, Glu, Arg, Gly, and Gln substitutions permitted binding, whereas Lys or Trp substitutions abrogated recognition. Since site-directed mutations to amino acids involve positive (Arg), negative (Glu), and neutral (Asn, Ala, Gly, and Gln) amino acids, hydrophilic (Glu, Arg, and Gln), hydrophobic (Ala), hydrogen bond related (Asn and Gln), and non-hydrogen bond related (Gly and Ala) mutations did not affect mAb D1 binding. Thus, electrostatic interactions, hydrogen bonding potential, and hydrophobic properties may not significantly contribute to mAb D1 binding affinity. On the contrary, mAb D1 failed to bind to Trp (rigid indole ring side chain) and Lys (–(CH_2_)_4_–NH_3_^+^ side chain with fixed ε-amino direction), suggesting that steric hindrance of the side chain may prevent the binding of mAb D1.

### Structure prediction

**Figure 6:**
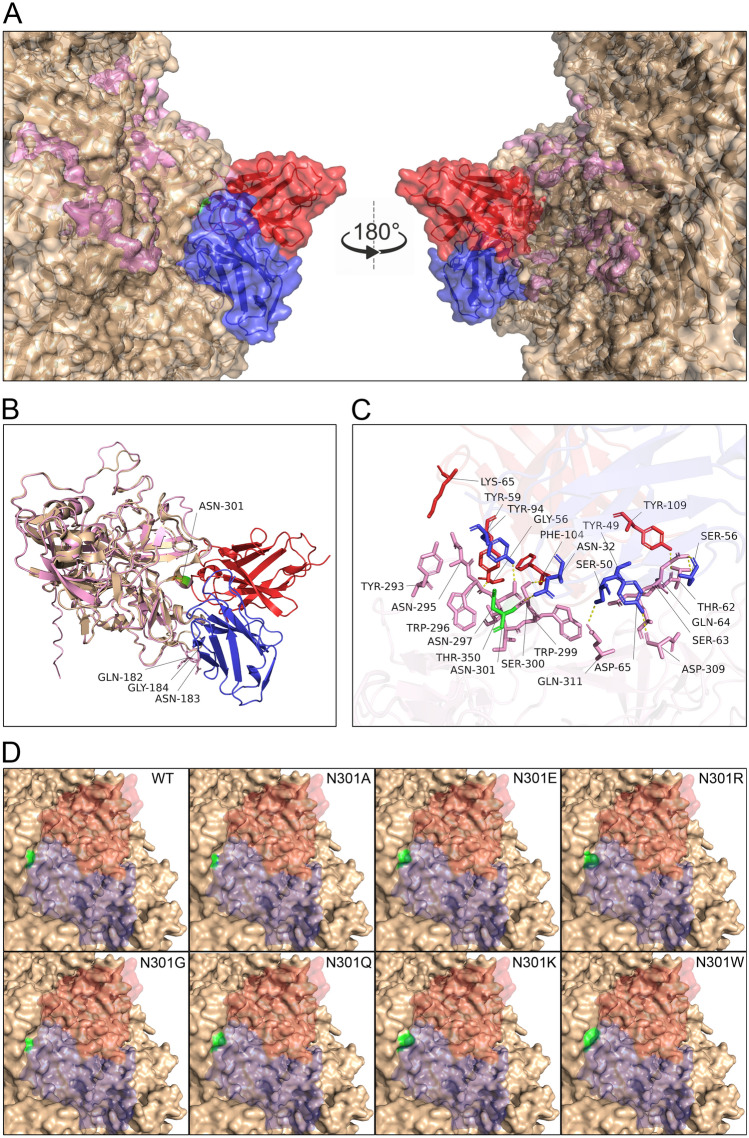
**The 3D structure of NP5–VP3–D1 binding complex.** The binding complex was predicted by AlphaFold 3, aligned to the goose parvovirus capsid (PDB ID: 9ME0), and presented as surface (**A**) and cartoon (**B**). The relevant residues combined with the interface (**C**). The 3D structure of different site-directed mutants of the aa301 of NP5 VP3 (**D**). The goose parvovirus capsid (PDB ID: 9ME0) is colored wheat, NP5 VP3 is colored pink, the variable region of the mAb D1 heavy chain is colored red, the variable region of the mAb D1 light chain is colored blue, and the residue of aa301 is colored green. The yellow dotted lines mark hydrogen bonds.

All 20 models of pTM + ipTM scores were 0.92–0.94, greater than the expected 0.75. The aa301 of VP3 were on the antigen–antibody interface of all models. Moreover, the antibodies of all models attach to the surface of the viral capsid by aligning to the goose parvovirus capsid (PDB ID: 9ME0). Thus, the structures of the 17 models were very similar, and the antigen–antibody interface contained CDR2 and CDR3 of VH and VL. The structures of the three remaining models were also very similar, but the antigen–antibody interface only contained CDR2 and CDR3 of VL and CDR3 of VH. Thus, the model with the highest chain_pair_iptm scores (0.86, 0.15, 0.15), (0.15, 0.85, 0.86), and (0.15, 0.86, 0.87) was selected as the WT of the NP5–VP3–D1 complex. The structure of the WT model aligned with 9ME0 (Figures 6A, B), with very high structural similarity (Figure 6B). However, the antigen–antibody binding interface of the predicted model exhibited structural differences at VP3 residues 182, 183, and 184 compared with the GPV capsid structure (PDB ID: 9ME0) (Figure 6B). The following residues may be involved in hydrogen bond formation: T62, S63, Q64, D65, N295, W296, N297, W299, S300, Q311, and T350 of VP3; Y59, G56, F104, and Y109 of VH; and N32, S50, S56, and Y94 of VL (Figure 6C). Figure 6D shows the models of site-directed mutations where the steric hindrance of the 301 residues of N301A and N301G is much smaller than that of WT. Moreover, N301E and N301Q were slightly raised but similar to the WT. However, N301R, N301K, and N301W were much more steric than WT.

### Molecular dynamics

#### RMSD analysis

**Figure 7:**
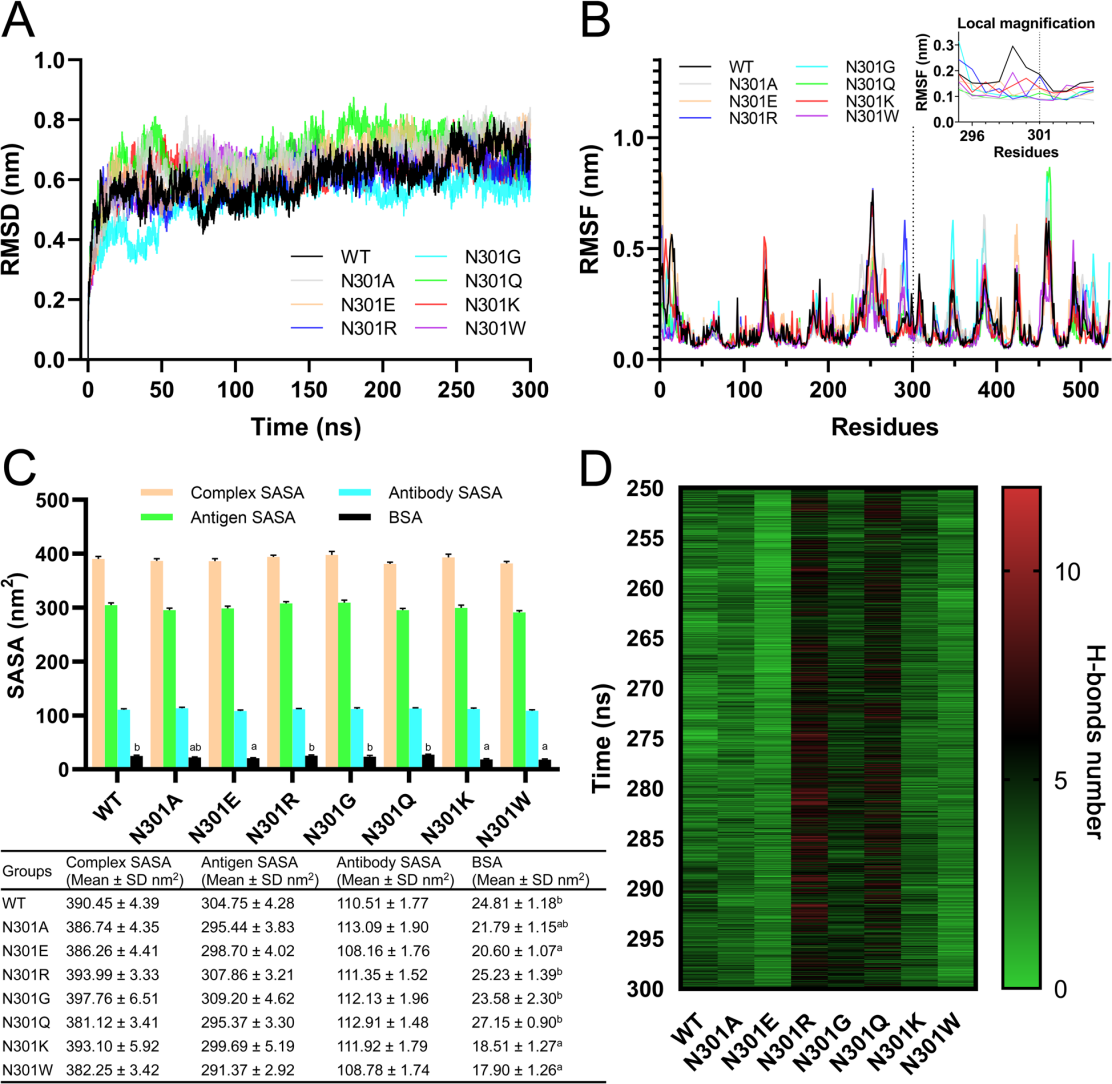
**The molecular dynamics of D1 ligand against the wild-type (WT) and mutants of NP5 VP3.** (**A**) Root mean square deviation (RMSD) and (**B**) root mean square fluctuation (RMSF) of VP3, relative to the initial conformation, for wild-type (WT) and mutant NP5 VP3 versus simulation time (ns) in production simulations with the D1 antibody variable region. (**C**) Buried surface area (BSA) during the 200–250 ns of the simulation time. Statistical analysis was performed using one-way ANOVA with Tukey’s post hoc test. Groups sharing the same superscript letter in the BSA column are not significantly different (*p* > 0.05). Differences are highly significant (*p* < 0.01) or significant (0.01 < *p* < 0.05) otherwise. (**D**) Heat map of the hydrogen bond network between VP3 and D1 during the 200–250 ns of the simulation time.

The RMSD profiles of all simulated systems (WT and mutant NP5) exhibited similar trends during the 50–300 ns production phase of the GROMACS molecular dynamics simulations (Figure 7A). An initial rapid increase in RMSD values (0–50 ns) indicated structural rearrangement from the starting conformation. Subsequently, the systems reached equilibrium with RMSD fluctuations centered around 0.6 nm, suggesting stable conformational sampling.

#### RMSF analysis

The backbone RMSF profiles of VP3 residues revealed distinct, flexible patterns correlated with antibody recognition since VP3 has many flexible regions. The main object evaluated by RMSF are residues near the antigen–antibody binding interface (Figure 6C). As a result (Figure 7B), there was no significant RMSD difference among each group within the residue range of aa62–65. However, the RMSD values displayed a clear descending order in the aa288–294 residue range. N301R showed the highest value, followed by N301G, with a moderately lower value.

The next tier consisted of comparable levels of N301E and N301A, while N301W slightly decreased. The WT, N301K, and N301Q had the lowest and nearly identical values. Additionally, local magnification around aa301 (Figure 7B) revealed the highest conformational flexibility of the WT, consistent with its optimal binding capacity. Next, N301K, N301R, and N301W showed different intermediate rigidities with localized fluctuations, as N301W showed the highest RMSD at aa299, N301K at aa300, and N301R at aa301. N301A, N301E, N301G, and N301Q maintained the most constrained structural profile. Lys, Arg, and Trp, have large side chains, and Ala, Glu, Gly, and Gln have small side chains, so the RMSF seems to be related to the side chain of aa301. Notably, N301R displayed significantly higher flexibility at position 301 and induced enhanced mobility in aa288–294 despite sharing similar steric hindrance with N301K/N301W due to the extended side chain of arginine (Figure 6D). This unique dynamic profile likely enables antibody D1 recognition of N301R but not N301K/N301W.

#### BSA analysis

The computed BSA values (Figures 7C, D) during the 200–250 ns of simulation time revealed significant differences among variants. The binding-competent N301Q exhibited the largest interface area (27.15 ± 0.9 nm^2^), followed by N301R (25.23 ± 1.39 nm^2^), WT (24.81 ± 1.18 nm^2^), N301G (23.58 ± 2.30 nm^2^), N301A (21.79 ± 1.15 nm^2^), and N301E (20.60 ± 1.07 nm^2^). In contrast, the non-binding mutants N301K (18.51 ± 1.27 nm^2^) and N301W (17.90 ± 1.26 nm^2^) showed highly significantly reduced BSA compared with N301Q, N301R, WT, and N301G (*p* < 0.01, one-way ANOVA with Tukey’s post hoc test), and significantly reduced BSA compared with N301A (0.01 < *p* < 0.05), consistent with experimental binding results. Additionally, N301Q showed highly significantly greater BSA than N301A and N301E (*p* < 0.01). No significant differences were observed between N301K, N301W, and N301E (*p* > 0.05).

#### H‑bonds analysis

The hydrogen bond analysis (Figure 7E) revealed a different pattern. N301R, N301Q, N301G, and N301K had more abundant hydrogen bond networks than WT and other mutations, while N301E was the least. N301R and N301G showed the highest H-bond occupancy (100%), followed by N301Q (99.98%), N301K (99.64%), N301A (99.58%), N301W (98.18%), WT (96.56%), and N301E (91.12%). Unique residue pairs analysis showed that WT formed the most extensive network (56 unique pairs), followed by N301R (48 unique pairs), N301G (45 unique pairs), N301Q (39 unique pairs), N301K (37 pairs), and N301E (32 pairs). However, despite different binding phenotypes, N301A/W showed identical pair counts (24 pairs). The VP3 residues (T62, S63, and Q64) were involved in hydrogen-bond networks in all the six D1-binding models (WT and mutants N301A/E/R/G/Q). D65, Q182, Y293, N295, W296, and S300 were involved in five out of the six models, and N183, N181, N297, W299, A351, S348, T349, and T350 were involved in four out of the six models.

#### MM/PBSA calculations

**Figure 8:**
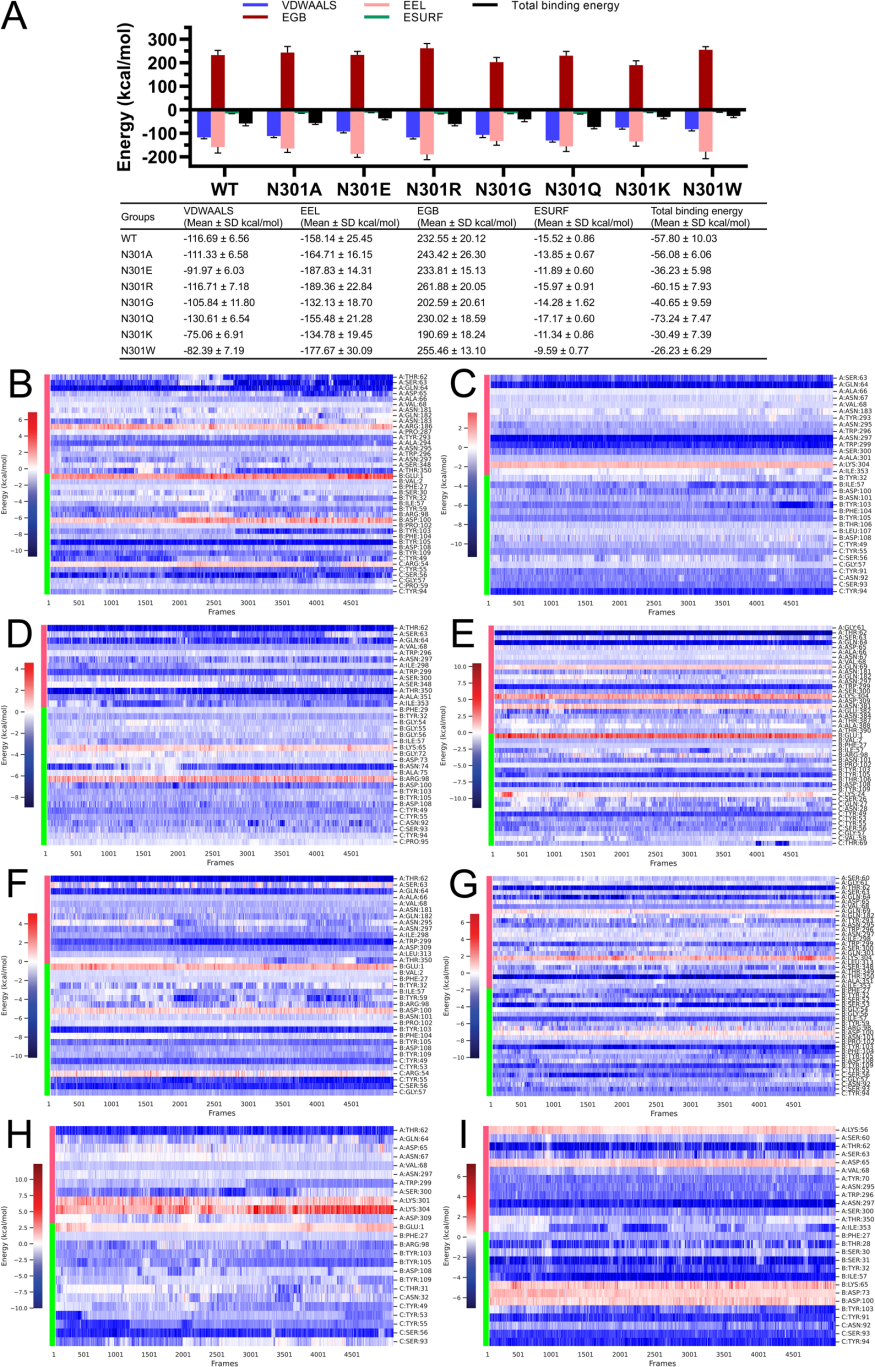
**Free energy estimation of the D1 ligand against wild-type (WT) and mutant NP5 VP3.** Free energy estimation data (**A**). The residue-wise energy decomposition of the WT (**B**) and N301A (**C**), N301E (**D**), N301R (**E**), N301G (**F**), N301Q (**G**), N301K (**H**), and N301W (**I**) mutants.

The binding free energy was calculated using the MM/PBSA method, revealing distinct thermodynamic profiles between the binding-competent (WT, N301A/E/R/G/Q) and nonbinding (N301K/W) variants (Figure 8A). N301Q exhibited the strongest binding affinity (Δ*G*_bind_ = −73.24 ± 7.47 kcal/mol), followed by N301R (Δ*G*_bind_ = −60.15 ± 7.93 kcal/mol), WT (Δ*G*_bind_ = −57.80 ± 10.03 kcal/mol), N301A (−56.08 ± 6.06), N301G (Δ*G*_bind_ = −40.65 ± 9.59 kcal/mol), and N301E (−36.23 ± 5.98). In contrast, the disruptive mutants (N301K and N301W) showed significantly low total energies (−30.49 ± 7.39 and −26.23 ± 6.29 kcal/mol, respectively), aligning with experimental binding failure.

Residue-wise energy decomposition identified critical binding hotspots across variants. For the wild-type (Figure 8B) and mAb D1-binding mutants (N301A/E/R/G/Q; Figures 8C–G), the following VP3 residues demonstrated strong binding energy contributions (> 2 kcal/mol): T62, S63, Q64, V68, N181, Y293, A294, N295, W296, N297, I298, W299, S300, K304, D309, S348, T350, A351, I353, E382, and N384. Among them, Q64 (in WT and N301A/E/R/G/Q), T62 (in WT and N301E/R/G/Q), W299 (in N301A/E/R/G/Q), and T350 (in WT and N301E/Q) exhibited more frequent binding (> 80%). T62, S63, Q64, N297, W299, and T350 maintained the key interactions (> 4 kcal/mol), while the nonbinding variants showed distinct disruption patterns. The N301K mutant (Figure 8H) introduced lysine residues and created and enhanced repulsion (K301: 1.37 ± 1.41 kcal/mol; K304: 3.1 ± 1.91 kcal/mol), while concurrently weakening the Q64 interaction (−1.86 ± 1.19 kcal/mol) and inducing > 4 Å spatial rearrangements. These combined effects substantially reduced the receptor (antigen) binding affinity (Δ*G*_receptor_ = −12.11 kcal/mol) compared with the WT (−33.27 kcal/mol). Moreover, the N301W variant (Figure 8I) demonstrated a multifaceted inhibition mechanism beyond its moderate impact on antigen binding (Δ*G*_receptor_ = −18.94 kcal/mol). This mutation uniquely induced repulsive interactions with multiple VH chain residues (K65/D73/D100, Δ*G* > 0), suggesting an allosteric disruption of antibody framework stability that extends beyond direct binding site interference.

### Role of VP3 aa65 and aa296 in mAb D1 Binding to GPV and MDPV

**Figure 9:**
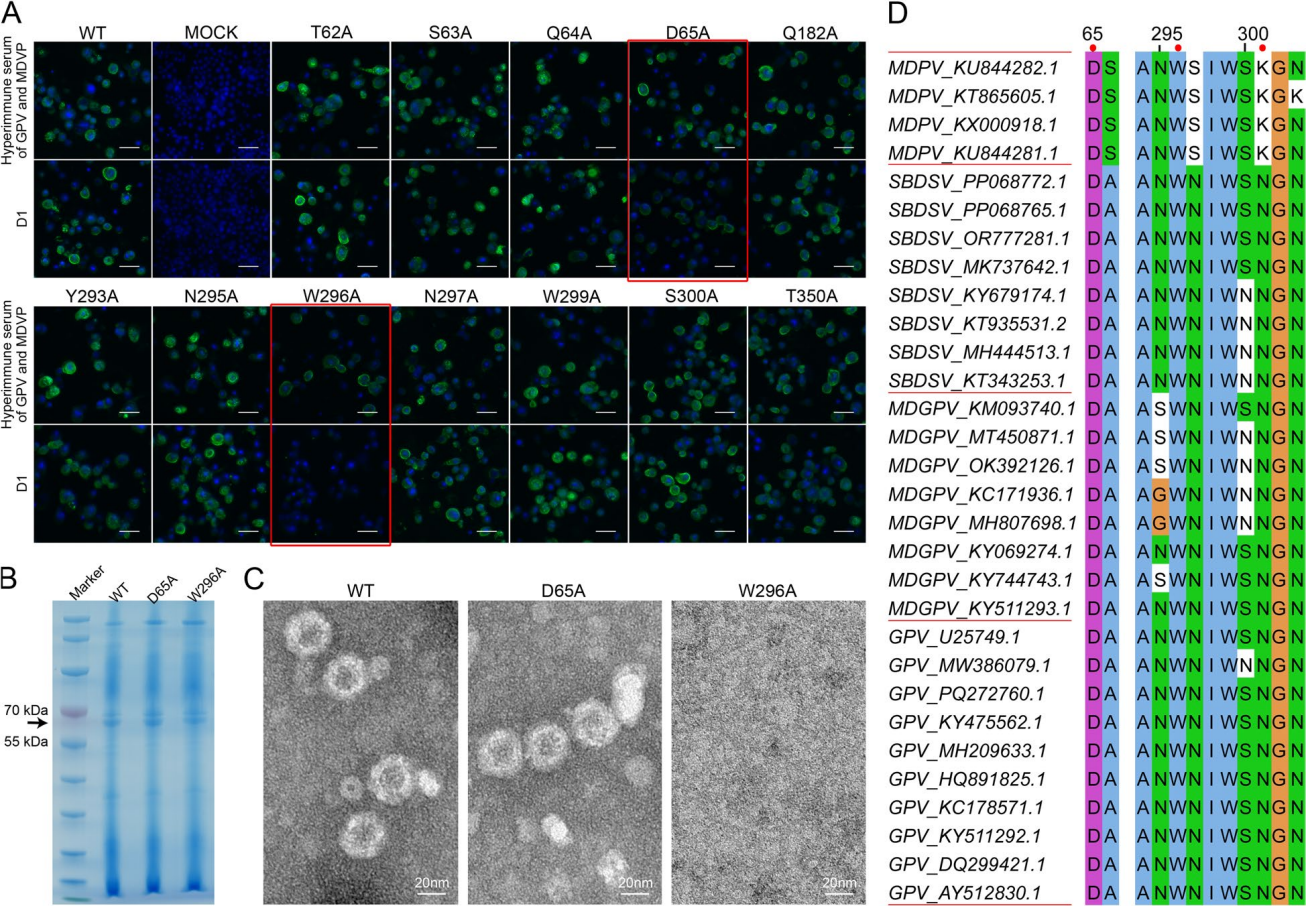
**Validation of the relevant residues involved in binding.** Indirect immunofluorescence results of alanine mutations in related residues of VP3 (**A**). Monoclonal antibody D1 and mouse hyperimmune serum were used as primary antibodies, and FITC-conjugated goat anti-mouse IgG antibody was used as the secondary antibody. SDS-PAGE of whole proteins of wild-type (WT) NP5 VP3 and alanine mutants expressed by the baculovirus expression system (**B**). The transmission electron microscope images of WT NP5 VP3 and alanine mutation samples were prepared by differential centrifugation (**C**). Amino acid composition of relevant residues in MDPV, SBDSV, MDGPV, and GPV strains (**D**). Scale bars (white), 50 μm (**A**, unlabeled) and 20 nm (**C**, labeled).

The important residues involved in binding energy contributions and hydrogen-bond networks in NP5 WT and mAb binding-mutants (N301A/E/R/G/Q), viz. T62, S63, Q64, D65, Q182, Y293, N295, W296, N297, W299, S300, and T350, were selected for alanine scanning. All mutants other than D65M and W296M did not affect mAb D1 binding (Figure 9A). In particular, the W296M mutation reduced the binding of mAb D1 and hyperimmune serum. WT, D65M, and W296M were analyzed by SDS-PAGE to ensure that proteins were expressed correctly. The results were bands of similar thickness (63.4 kDa) (Figure 9B). Furthermore, W296M failed to self-assemble into VLP, while WT and D65M assembled normally (Figure 9C). Therefore, aa65 and aa296 of NP5 VP3 may be related to the binding of the D1 monoclonal antibody, and aa296 may be important for maintaining the overall epitope structure. Comparative analysis of VP3 residues across viral strains (Figure 9D) revealed strict conservation at positions 65 (D) and 296 (W) in all the examined variants. Notably, residue 301 exhibited strain-specific variation: K in MDPV versus N in GPV, SBDSV, and the MDGPV chimera.

## Discussion

The precise identification of mAb-recognized viral epitopes holds significant implications for controlling parvoviruses in waterfowl. Monoclonal antibody D1, an IgG1 subtype monoclonal antibody with a significantly higher neutralization titer (9 log_2_) to GPV, was produced from hybridoma cells following a standard long-term immunization protocol. This antibody demonstrates high specificity, elevated titer, and stable passage, indicating it targets a dominant antigenic epitope that elicits a robust immune response in GPV-related parvoviruses. Long-term and repeated immunization induces the production of high-affinity antibodies targeting dominant epitopes [34, 35], which are critical for disease prevention and control. Characterization of the epitope recognized by monoclonal antibody D1 supports classification of viral serotypes, facilitates monitoring of viral mutations, and informs vaccine strain selection, as mutations in the epitope and its surrounding regions may cause immune escape, necessitating vigilant monitoring. This knowledge may enable development of serotype identification products, such as ELISA-based diagnostic assays and colloidal gold strip tests, enhancing strategies for disease prevention and control.

This study selected GPV NP5 strain and MDPV P1 strain for epitope mapping on the basis of their genetic and epidemiological representativeness and their classic and prevalent characteristics, aiming to investigate epitope differences. For GPV, NP5 exhibits 99.7% homology with classic strains and ≥ 92.8% with other prevalent strains, and its wide circulation in waterfowl populations in China, associated with significant outbreaks, supports its relevance for diagnostic and vaccine development [36]. For MDPV, P1, an attenuated derivative of the classic P strain, shows ≥ 94.9% homology with reported strains [37]. To characterize the epitope, VP3 protein served as the antigen, with TEM confirming that amino acid substitutions between GPV and MDPV do not impair VLP assembly (Figure 3). Although the native parvovirus capsid comprises VP1, VP2, and VP3, VLPs formed solely by VP2 or VP3 are established as feasible models for studying capsid structure [30, 38]. Furthermore, experimental evidence verified that monoclonal antibody D1 recognizes a single VP3 subunit (Figure 3), supporting the use of a single VP3 sequence for structural prediction and molecular dynamics simulations.

Our findings highlight VP3-301 as a critical and conserved residue for mAb D1 binding, which consistently presenting as Lys in MDPV but as Asn in GPV, MDGPV, and SBDSV. The site-directed mutagenesis at residue 301 was designed to probe the molecular basis of differential binding of the mAb D1, using a resource-efficient approach. Substitutions with Glu, Arg, Trp, Gly, Ala, and Gln were chosen to test diverse physicochemical properties (charge, hydrophobicity, steric hindrance, and hydrogen bonding), as these factors often govern epitope–antibody interactions [39]. This targeted strategy, guided by the AlphaFold 3 prediction of residue 301 as surface-exposed, confirmed steric hindrance as the primary determinant of mAb D1 binding, validated by immunofluorescence assays, molecular dynamics simulations, and PyMOL visualization. While additional residues (e.g., proline for rigidity or cysteine for disulfide potential) could further elucidate structural effects, resource constraints limited our scope, and the current substitutions sufficiently identified the critical epitope, aligning with the study’s objectives.

Further analysis identified additional participating residues (65, 296), collectively delineating the mAb D1 epitope. Interestingly, the W296A mutation disrupted VLP self-assembly, underscoring its structural importance beyond epitope engagement. Conversely, alanine substitutions at other predicted sites did not abrogate binding; however, this does not preclude their participation in the interface, as single-point mutations may be insufficient to disrupt complex interactions. Definitive epitope mapping requires additional structural validation (e.g., crystallography), and the congruence between computational predictions and experimental data establishes this integrated approach as a strategically valuable methodology.

Given the absence of structural epitope studies on waterfowl parvoviruses, our work represents the first investigation in this area. Previous studies identified linear B-cell epitopes on VP3, such as ^438^LHNPPP^443^ [40] and ^82^FxRFHxH^88^ [25], and VP1 regions 35–71, 123–198, and 423–444 [41]. To place our findings in context, literature on adeno-associated virus serotype 5 (AAV5), a *Parvoviridae* family member with ~ 59% VP3 amino acid similarity and structural homology to waterfowl parvoviruses, indicates that VP3 residues 65, 296, and 301 correspond to AAV5 VP residues 258, 486, and 491, contact residues for monoclonal antibody 3C5 (IgG3) [42]. This structural correspondence supports the robustness of our epitope mapping, reinforcing the significance of residues 65, 296, and 301 in defining the monoclonal antibody D1 epitope.

AlphaFold 3 demonstrated substantial predictive credibility. When provided solely with the antigenic VP3 sequence and antibody variable region sequences, all 20 independent prediction runs generated models encompassing residue 301. Structural alignment revealed a high similarity between these predicted models and the experimentally determined viral capsid structure (Figure 6B). The molecular docking model prediction results were unsatisfactory. AlphaFold 3 demonstrates superior performance (47% success rate) in antigen–antibody docking scenarios compared with template-based docking tools (35%) [43]. Thus, we performed molecular dynamics simulations on the AlphaFold 3 models to evaluate these predictions computationally. The calculated BSA and binding free energy supported the IF binding assays where mAb D1 bound to WT NP5 VP3 and N301A/E/R/G/Q mutants but failed to recognize N301K/W mutants. K301 likely induces steric hindrance, explaining the inability of mAb D1 to bind MDPV. Although R301 and K/W301 possess extended side chains capable of steric interference, R301 exhibited greater conformational flexibility (Figure 7B), facilitating stronger hydrogen bonding (Figure 7D) and generating more favorable electrostatic interactions (Figure 8A). While IF confirmed that mAb D1 binds to N301E and N301G mutants, the attenuated binding affinity likely results from changes in electrostatic potential (Figure 8A), warranting further experimental quantification.

This integrated approach combines AlphaFold 3-based structural prediction, molecular dynamics simulations, and experimental validation and has identified residues 301, 65, and 296 of VP3 as critical components of D1. Position 301 represents a conserved divergent site (Asn in GPV/MDGPV/SBDSV versus Lys in MDPV), and structural analyses suggest that the Lys at this position in MDPV likely induces steric hindrance, explaining the inability of D1 to bind MPV.

## Supplementary Information

**Additional file 1. Primers used in the study.**

## Data Availability

The datasets used and/or analyzed during the current study are available from the corresponding author on reasonable request.
