# Supplementary material for: Residue 301-dependent epitope mapping reveals the molecular basis for GPV/MDPV serotype discrimination by neutralizing monoclonal antibody D1
Source: Vet Res. 2025 Oct 16;56:196. doi: 10.1186/s13567-025-01625-6 (PMC12532459; doi:10.1186/s13567-025-01625-6)
Supplement: Supplementary file 1 — Additional file 1. Primers used in the study. [file 13567_2025_1625_MOESM1_ESM.docx]

| **Primer Name** | **Sequence (5’–3’)** | **Target** | **Purpose** | **Template/ Vector** | **Carrier vector** | **Section/Figure** |
| --- | --- | --- | --- | --- | --- | --- |
| HTB-NP5F | tttcagggcgccatgggatccATGGCAGAGGGAGGAGGCG | VP3 full gene | Amplification | GPV NP5 | pFastBac HTB | 3.1 / Fig. 1 |
| HTB-NP5R | gctctagattcgaaagcggccgcTTACAGATTTTGAGTTAGATATCTGGTTCC |  |  |  |  |  |
| HTB-M3F | GGGCGCCATGGGATCCATGGCAGAGGGAGGAAGC | VP3 full gene | Amplification | MDPV P1 | pFastBac HTB | 3.1 / Fig. 1 |
| HTB-M3R | GATTCGAAAGCGGCCGCTTACAGATTCTGAGTCAAATACCTG |  |  |  |  |  |
| G1G2M3-F1 | atattttaggtttagctaaaGACCCCTACAGGTCAGGCAGT | VP3 Region 3 (342-543 aa) | Amplification | pFastBac HTB - MDPV P1 | pFastBac HTB | 3.3 / Fig. 3 |
| G1G2M3-R1 | ctagattcgaaagcggccgcTTACAGATTCTGAGTCAAATACCTGGT |  |  |  |  |  |
| G1G2M3-F2 | GCGGCCGCTTTCGAATCT | pFastBac HTB - GPV NP5 except VP3 Region 3 | Amplification | pFastBac HTB - GPV NP5 | pFastBac HTB | 3.3 / Fig. 3 |
| G1G2M3-R2 | TTTAGCTAAACCTAAAATATTTTGGGC |  |  |  |  |  |
| G1M2M3-F1 | ttcagggcgccatgggatccATGGCAGAGGGAGGAGGCG | VP3 Region 1 (1-229 aa) | Amplification | pFastBac HTB - GPV NP5 | pFastBac HTB | 3.3 / Fig. 3 |
| G1M2M3-R1 | ttcattagcctgtctaaatcCTGTGAATGAGCGAACATGCTATG |  |  |  |  |  |
| G1M2M3-F2 | GATTTAGACAGGCTAATGAATCCTCTC | pFastBac HTB - MDPV P1 except VP3 Region 1 | Amplification | pFastBac HTB - MDPV P1 | pFastBac HTB | 3.3 / Fig. 3 |
| G1M2M3-R2 | GGATCCCATGGCGCCCTG |  |  |  |  |  |
| G1M2G3-F1 | gcatgttcgctcattcacagGATTTAGACAGGCTAATGAATCCTCTC | VP3 Region 2 (230-341) | Amplification | pFastBac HTB - MDPV P1 | pFastBac HTB | 3.3 / Fig. 3 |
| G1M2G3-R1 | ctgccagatctgtatggatcTTTTGCAATTCCTATTGTGTTTTGA |  |  |  |  |  |
| G1M2G3-F2 | GATCCATACAGATCTGGCAGCA | pFastBac HTB - GPV NP5 except VP3 Region 2 | Amplification | pFastBac HTB - GPV NP5 | pFastBac HTB | 3.3 / Fig. 3 |
| G1M2G3-R2 | CTGTGAATGAGCGAACATGCTATG |  |  |  |  |  |
| M1G2M3-F1 | gcatgttcgctcattcacagGACTTAGACAGGCTGATGAACCCC | VP3 Region 2 (230-341) | Amplification | pFastBac HTB - GPV NP5 | pFastBac HTB | 3.3 / Fig. 3 |
| M1G2M3-R1 | ctgcctgacctgtaggggtcTTTAGCTAAACCTAAAATATTTTGGGC |  |  |  |  |  |
| M1G2M3-F2 | GACCCCTACAGGTCAGGCAGT | pFastBac HTB - MDPV P1 except VP3 Region 2 | Amplification | pFastBac HTB - MDPV P1 | pFastBac HTB | 3.3 / Fig. 3 |
| M1G2M3-R2 | CTGTGAATGAGCGAACATGCTATG |  |  |  |  |  |
| M1G2G3-F1 | ttcagggcgccatgggatccATGGCAGAGGGAGGAAGCG | VP3 Region 1 (1-229 aa) | Amplification | pFastBac HTB - MDPV P1 | pFastBac HTB | 3.3 / Fig. 3 |
| M1G2G3-R1 | ttcatcagcctgtctaagtcCTGTGAATGAGCGAACATGCTATG |  |  |  |  |  |
| M1G2G3-F2 | GACTTAGACAGGCTGATGAACCCC | pFastBac HTB - GPV NP5 except VP3 Region 1 | Amplification | pFastBac HTB - GPV NP5 | pFastBac HTB | 3.3 / Fig. 3 |
| M1G2G3-R2 | GGATCCCATGGCGCCCTG |  |  |  |  |  |
| M1M2G3-F1 | acacaataggaattgcaaaaGATCCATACAGATCTGGCAGCA | VP3 Region 3 (342-543 aa) | Amplification | pFastBac HTB - GPV NP5 | pFastBac HTB | 3.3 / Fig. 3 |
| M1M2G3-R1 | ctagattcgaaagcggccgcTTACAGATTTTGAGTTAGATATCTGGTTCC |  |  |  |  |  |
| M1M2G3-F2 | GCGGCCGCTTTCGAATCT | pFastBac HTB - MDPV P1 except VP3 Region 3 | Amplification | pFastBac HTB - MDPV P1 | pFastBac HTB | 3.3 / Fig. 3 |
| M1M2G3-R2 | TTTTGCAATTCCTATTGTGTTTTGA |  |  |  |  |  |
| Q64P-F | AACCTCTccgGATGCAAATGTCCAGTATGCAGG | aa64 (Gln to Pro) | Mutagenesis | pFastBac HTB - GPV NP5 | pFastBac HTB | 3.3 / Fig. 4 |
| Q64P-R | TTGCATCcggAGAGGTTCCACTGGTAATCGCTT |  |  |  |  |  |
| A66S-F | GAACCTCTCAAGATagcAATGTCCAGTATGCAGGATACAGTACC | aa66 (Ala to Ser) | Mutagenesis | pFastBac HTB - GPV NP5 | pFastBac HTB | 3.3 / Fig. 4 |
| A66S-R | gctATCTTGAGAGGTTCCACTGGTAATCGCTT |  |  |  |  |  |
| V68T-F | GCAAATaccCAGTATGCAGGATACAGTACCCCC | aa68 (Val to Thr) | Mutagenesis | pFastBac HTB - GPV NP5 | pFastBac HTB | 3.3 / Fig. 4 |
| V68T-R | GCATACTGggtATTTGCATCTTGAGAGGTTCCAC |  |  |  |  |  |
| S108A-F | ACCCAAGgcgCTTAAATTCAAGATCTTCAATGTCCAA | aa108 (Ser to Ala) | Mutagenesis | pFastBac HTB - GPV NP5 | pFastBac HTB | 3.3 / Fig. 4 |
| S108A-R | ATTTAAGcgcCTTGGGTCTGATTCCCCAATGG |  |  |  |  |  |
| V140I-F | AattTTTACGGATGACGAGCATCAACTCCCGT | aa140 (Val to Ile) | Mutagenesis | pFastBac HTB - GPV NP5 | pFastBac HTB | 3.3 / Fig. 4 |
| V140I-R | CGTCATCCGTAAAaatTTGAATTGTTGACGTGAGATTGTTT |  |  |  |  |  |
| D144N-F | TACGGATaacGAGCATCAACTCCCGTATGTCC | aa144 (Asp to Asn) | Mutagenesis | pFastBac HTB - GPV NP5 | pFastBac HTB | 3.3 / Fig. 4 |
| D144N-R | GATGCTCgttATCCGTAAAGACTTGAATTGTTGACG |  |  |  |  |  |
| N183S-F | ACACCAACCAGagcGGAGCACGGTTCAATGACCG | aa183 (Asn to Ser) | Mutagenesis | pFastBac HTB - GPV NP5 | pFastBac HTB | 3.3 / Fig. 4 |
| N183S-R | TCCgctCTGGTTGGTGTGCATGGTGCAGTACC |  |  |  |  |  |
| T213S-F | TGAGTTCagcTTTGACTTTGAAGAAGTTCCTTTCC | aa213 (Thr to Ser) | Mutagenesis | pFastBac HTB - GPV NP5 | pFastBac HTB | 3.3 / Fig. 4 |
| T213S-R | AGTCAAAgctGAACTCAAAGTTGTTGCCTGTTCTT |  |  |  |  |  |
| D215E-F | gaaTTTGAAGAAGTTCCTTTCCATAGCATGTT | aa215 (Asp to Glu) | Mutagenesis | pFastBac HTB - GPV NP5 | pFastBac HTB | 3.3 / Fig. 4 |
| D215E-R | GGAACTTCTTCAAAttcAAATGTGAACTCAAAGTTGTTGCC |  |  |  |  |  |
| V239L-F | CCCCCTActgGATCAATACCTCTGGAATTTCAATGA | aa239 (Val to Leu) | Mutagenesis | pFastBac HTB - GPV NP5 | pFastBac HTB | 3.3 / Fig. 4 |
| V239L-R | ATTGATCcagTAGGGGGTTCATCAGCCTGTCT |  |  |  |  |  |
| N247S-F | GAATTTCagcGAGGTAGACAGCAGCAGAAATGC | aa247 (Asn to Ser) | Mutagenesis | pFastBac HTB - GPV NP5 | pFastBac HTB | 3.3 / Fig. 4 |
| N247S-R | CTACCTCgctGAAATTCCAGAGGTATTGATCCACT |  |  |  |  |  |
| D250N-F | TGAGGTAaacAGCAGCAGAAATGCTCAATTTAAA | aa250 (Asp to Asn) | Mutagenesis | pFastBac HTB - GPV NP5 | pFastBac HTB | 3.3 / Fig. 4 |
| D250N-R | TGCTGCTgttTACCTCATTGAAATTCCAGAGGTATT |  |  |  |  |  |
| S251G-F | GTAGACggcAGCAGAAATGCTCAATTTAAAAAGG | aa251 (Ser to Gly) | Mutagenesis | pFastBac HTB - GPV NP5 | pFastBac HTB | 3.3 / Fig. 4 |
| S251G-R | TTTCTGCTgccGTCTACCTCATTGAAATTCCAGAGG |  |  |  |  |  |
| Y265F-F | AAAGGGGCTtttGGCACCATGGGCCGCAATTG | aa265 (Tyr to Phe) | Mutagenesis | pFastBac HTB - GPV NP5 | pFastBac HTB | 3.3 / Fig. 4 |
| Y265F-R | GTGCCaaaAGCCCCTTTCACAGCCTTTTTAAA |  |  |  |  |  |
| T267A-F | TTATGGCgcgATGGGCCGCAATTGGCTGCCAG | aa267 (Thr to Ala) | Mutagenesis | pFastBac HTB - GPV NP5 | pFastBac HTB | 3.3 / Fig. 4 |
| T267A-R | GGCCCATcgcGCCATAAGCCCCTTTCACAGCC |  |  |  |  |  |
| F278L-F | ACCTAAActgCTGGACCAGAGAGTTAGGGCCT | aa278 (Phe to Leu) | Mutagenesis | pFastBac HTB - GPV NP5 | pFastBac HTB | 3.3 / Fig. 4 |
| F278L-R | GGTCCAGcagTTTAGGTCCTGGCAGCCAATTG |  |  |  |  |  |
| N297S-F | AAACTGGagcATCTGGAGTAATGGAAACAAGGTTAA | aa297 (Asn to Ser) | Mutagenesis | pFastBac HTB - GPV NP5 | pFastBac HTB | 3.3 / Fig. 4 |
| N297S-R | TCCAGATgctCCAGTTTGCATAATTATCTGTTCCG |  |  |  |  |  |
| N301K-F | CTGGAGTaaaGGAAACAAGGTTAATTTGAAGGACA | aa301 (Asn to Lys) | Mutagenesis | pFastBac HTB - GPV NP5 | pFastBac HTB | 3.3 / Fig. 4 |
| N301K-R | TGTTTCCtttACTCCAGATGTTCCAGTTTGCAT |  |  |  |  |  |
| N306F-F | TtttTTGAAGGACAGGCAGTACCTCCTGCAAC | aa306 (Asn to Phe) | Mutagenesis | pFastBac HTB - GPV NP5 | pFastBac HTB | 3.3 / Fig. 4 |
| N306F-R | GCCTGTCCTTCAAaaaAACCTTGTTTCCATTACTCCAGATG |  |  |  |  |  |
| Q311E-F | AGGACAGGgaaTACCTCCTGCAACTTGGACCTG | aa311 (Gln to Glu) | Mutagenesis | pFastBac HTB - GPV NP5 | pFastBac HTB | 3.3 / Fig. 4 |
| Q311E-R | GAGGTAttcCCTGTCCTTCAAATTAACCTTGTTT |  |  |  |  |  |
| S320A-F | TGTAgcgGCTACTCACACAAAAGCAGAGGCTT | aa320 (Ser to Ala) | Mutagenesis | pFastBac HTB - GPV NP5 | pFastBac HTB | 3.3 / Fig. 4 |
| S320A-R | TGTGAGTAGCcgcTACAGGTCCAAGTTGCAGGAGG |  |  |  |  |  |
| A321T-F | CTGTATCAaccACTCACACAAAAGCAGAGGCTTC | aa321 (Ala to Thr) | Mutagenesis | pFastBac HTB - GPV NP5 | pFastBac HTB | 3.3 / Fig. 4 |
| A321T-R | GTGAGTggtTGATACAGGTCCAAGTTGCAGGA |  |  |  |  |  |
| E327Q-F | CAAAAGCAcaaGCTTCCAGCATCCCAGCCCAA | aa327 (Glu to Gln) | Mutagenesis | pFastBac HTB - GPV NP5 | pFastBac HTB | 3.3 / Fig. 4 |
| E327Q-R | GGAAGCttgTGCTTTTGTGTGAGTAGCTGATACAG |  |  |  |  |  |
| L337I-F | GCCCAAAATATTattGGTTTAGCTAAAGATCCATACAGATCTG | aa337 (Leu to Ile) | Mutagenesis | pFastBac HTB - GPV NP5 | pFastBac HTB | 3.3 / Fig. 4 |
| L337I-R | CCaatAATATTTTGGGCTGGGATGCTGGAAGC |  |  |  |  |  |
| L339I-F | GGTattGCTAAAGATCCATACAGATCTGGCAG | aa339 (Leu to Ile) | Mutagenesis | pFastBac HTB - GPV NP5 | pFastBac HTB | 3.3 / Fig. 4 |
| L339I-R | GGATCTTTAGCaatACCTAAAATATTTTGGGCTGGGA |  |  |  |  |  |
| T349S-F | AGCagcACAGCAGGAATAAGTGATGTTATGGT | aa349 (Thr to Ser) | Mutagenesis | pFastBac HTB - GPV NP5 | pFastBac HTB | 3.3 / Fig. 4 |
| T349S-R | ATTCCTGCTGTgctGCTGCCAGATCTGTATGGATCTT |  |  |  |  |  |
| T350L-F | AGCACTctgGCAGGAATAAGTGATGTTATGGTCA | aa364 (Val to Ile) | Mutagenesis | pFastBac HTB - GPV NP5 | pFastBac HTB | 3.3 / Fig. 4 |
| T350L-R | ATTCCTGCcagAGTGCTGCCAGATCTGTATGGAT |  |  |  |  |  |
| K373R-F | TAGGGTGGcgcCCATATGGCAAGACTGTAACGAAT | aa373 (Lys to Arg) | Mutagenesis | pFastBac HTB - GPV NP5 | pFastBac HTB | 3.3 / Fig. 4 |
| K373R-R | ATATGGgcgCCACCCTACGCCGTTTGTAGGTG |  |  |  |  |  |
| S391N-F | TCCTACGaacTCAGATCTTGATGTTCCTGGAGC | aa391 (Ser to Asn) | Mutagenesis | pFastBac HTB - GPV NP5 | pFastBac HTB | 3.3 / Fig. 4 |
| S391N-R | GATCTGAgttCGTAGGAGCTGTAGTAGTGTTTTGTTC |  |  |  |  |  |
| S392A-F | TACGAGTgcgGATCTTGATGTTCCTGGAGCTTTAC | aa392 (Ser to Ala) | Mutagenesis | pFastBac HTB - GPV NP5 | pFastBac HTB | 3.3 / Fig. 4 |
| S392A-R | CAAGATCcgcACTCGTAGGAGCTGTAGTAGTGTTTTG |  |  |  |  |  |
| D395Q-F | AGATCTTcagGTTCCTGGAGCTTTACCAGGAA | aa395 (Asp to Gln) | Mutagenesis | pFastBac HTB - GPV NP5 | pFastBac HTB | 3.3 / Fig. 4 |
| D395Q-R | CAGGAACctgAAGATCTGAACTCGTAGGAGCTGTAG |  |  |  |  |  |
| P64Q-F | GGAACAAACcaaGACTCAAATACCCAATATGCTGGA | aa64 (Pro to Gln) | Mutagenesis | pFastBac HTB - MDPV P1 | pFastBac HTB | 3.3 / Fig. 4 |
| P64Q-R | GAGTCttgGTTTGTTCCGCTTGTGATGGCTTT |  |  |  |  |  |
| S66A-F | ACCCAGACgcaAATACCCAATATGCTGGATACAGCA | aa66 (Ser to Ala) | Mutagenesis | pFastBac HTB - MDPV P1 | pFastBac HTB | 3.3 / Fig. 4 |
| S66A-R | GGTATTtgcGTCTGGGTTTGTTCCGCTTGTGA |  |  |  |  |  |
| T68V-F | CCAGACTCAAATgtcCAATATGCTGGATACAGCACCCC | aa68 (Thr to Val) | Mutagenesis | pFastBac HTB - MDPV P1 | pFastBac HTB | 3.3 / Fig. 4 |
| T68V-R | TGgacATTTGAGTCTGGGTTTGTTCCGCTTGT |  |  |  |  |  |
| A108S-F | GACCGAAAtctCTCAAATTCAAGATATTCAATATGCAAG | aa108 (Ala to Ser) | Mutagenesis | pFastBac HTB - MDPV P1 | pFastBac HTB | 3.3 / Fig. 4 |
| A108S-R | TTTGAGagaTTTCGGTCTAATCCCCCAATGGT |  |  |  |  |  |
| I140V-F | AATCCAGgtcTTCACGGATAATGAACACCAGC | aa140 (Ile to Val) | Mutagenesis | pFastBac HTB - MDPV P1 | pFastBac HTB | 3.3 / Fig. 4 |
| I140V-R | CCGTGAAgacCTGGATTGTAGAGGTAAGGTTGTTAGC |  |  |  |  |  |
| N144D-F | TCACGGATgacGAACACCAGCTGCCCTATGTTC | aa144 (Asn to Asp) | Mutagenesis | pFastBac HTB - MDPV P1 | pFastBac HTB | 3.3 / Fig. 4 |
| N144D-R | GTGTTCgtcATCCGTGAATATCTGGATTGTAGAGG |  |  |  |  |  |
| S183N-F | CAACCAGaatGGAGCTGGATTCAATGACAGAAG | aa183 (Ser to Asn) | Mutagenesis | pFastBac HTB - MDPV P1 | pFastBac HTB | 3.3 / Fig. 4 |
| S183N-R | CAGCTCCattCTGGTTGGTGTGCATTGTGCAG |  |  |  |  |  |
| S213T-F | CacaTTTGAGTTTGAAGAAGTTCCCTTCCATA | aa213 (Ser to Thr) | Mutagenesis | pFastBac HTB - MDPV P1 | pFastBac HTB | 3.3 / Fig. 4 |
| S213T-R | CTTCAAACTCAAAtgtGAATTCAAAATTATTCCCTGTTCTCA |  |  |  |  |  |
| E215D-F | TCAGTTTTgacTTTGAAGAAGTTCCCTTCCATAGC | aa215 (Glu to Asp) | Mutagenesis | pFastBac HTB - MDPV P1 | pFastBac HTB | 3.3 / Fig. 4 |
| E215D-R | TTCAAAgtcAAAACTGAATTCAAAATTATTCCCTG |  |  |  |  |  |
| L239V-F | TCCTCTCgtgGATCAGTACCTGTGGAATTTCTCTGA | aa239 (Leu to Val) | Mutagenesis | pFastBac HTB - MDPV P1 | pFastBac HTB | 3.3 / Fig. 4 |
| L239V-R | ACTGATCcacGAGAGGATTCATTAGCCTGTCTAAATC |  |  |  |  |  |
| S247N-F | GTGGAATTTCaatGAGGTTAATGGTGGCAGGAATG | aa247 (Ser to Asn) | Mutagenesis | pFastBac HTB - MDPV P1 | pFastBac HTB | 3.3 / Fig. 4 |
| S247N-R | CCTCattGAAATTCCACAGGTACTGATCTAGGA |  |  |  |  |  |
| N250D-F | TCTCTGAGGTTgacGGTGGCAGGAATGCACAGTT | aa250 (Asn to Asp) | Mutagenesis | pFastBac HTB - MDPV P1 | pFastBac HTB | 3.3 / Fig. 4 |
| N250D-R | ACCgtcAACCTCAGAGAAATTCCACAGGTACT |  |  |  |  |  |
| G251S-F | GGTTAATagcGGCAGGAATGCACAGTTCAAAA | aa251 (Gly to Ser) | Mutagenesis | pFastBac HTB - MDPV P1 | pFastBac HTB | 3.3 / Fig. 4 |
| G251S-R | TCCTGCCgctATTAACCTCAGAGAAATTCCACAGG |  |  |  |  |  |
| F265Y-F | AGGAGCAtatGGTGCAATGGGGAGAAATTGGC | aa265 (Phe to Tyr) | Mutagenesis | pFastBac HTB - MDPV P1 | pFastBac HTB | 3.3 / Fig. 4 |
| F265Y-R | TTGCACCataTGCTCCTTTCACAGCTTTCTTG |  |  |  |  |  |
| A267T-F | ATTTGGTaccATGGGGAGAAATAGGCTTCCAG | aa267 (Ala to Thr) | Mutagenesis | pFastBac HTB - MDPV P1 | pFastBac HTB | 3.3 / Fig. 4 |
| A267T-R | TCCCCATggtACCAAATGCTCCTTTCACAGCT |  |  |  |  |  |
| L278F-F | ACCCAAAttcCTAGACCAAAGGGTAAGAGCATACC | aa278 (Leu to Phe) | Mutagenesis | pFastBac HTB - MDPV P1 | pFastBac HTB | 3.3 / Fig. 4 |
| L278F-R | GGTCTAGgaaTTTGGGTCCTGGAAGCCTATTT |  |  |  |  |  |
| S297N-F | GAACTGGaacATCTGGAGTAAAGGAAACAAAGTTTTT | aa297 (Ser to Asn) | Mutagenesis | pFastBac HTB - MDPV P1 | pFastBac HTB | 3.3 / Fig. 4 |
| S297N-R | TCCAGATgttCCAGTTCGCATAGTTATCTGTTCC |  |  |  |  |  |
| K301N-F | CTGGAGTaatGGAAACAAAGTTTTTCTTAAAGACAGAG | aa301 (Lys to Asn) | Mutagenesis | pFastBac HTB - MDPV P1 | pFastBac HTB | 3.3 / Fig. 4 |
| K301N-R | TGTTTCCattACTCCAGATTGACCAGTTCGCA |  |  |  |  |  |
| F306N-F | GTTaatCTTAAAGACAGAGAGTATCTACTGCAACC | aa306 (Phe to Asn) | Mutagenesis | pFastBac HTB - MDPV P1 | pFastBac HTB | 3.3 / Fig. 4 |
| F306N-R | CTGTCTTTAAGattAACTTTGTTTCCTTTACTCCAGATTG |  |  |  |  |  |
| E311Q-F | CAGAcagTATCTACTGCAACCAGGTCCAGTAGC | aa311 (Glu to Gln) | Mutagenesis | pFastBac HTB - MDPV P1 | pFastBac HTB | 3.3 / Fig. 4 |
| E311Q-R | GCAGTAGATActgTCTGTCTTTAAGAAAAACTTTGTTTCCT |  |  |  |  |  |
| A320S-F | GGTCCAGTAtcaACTACACATACAGAAGATCAGGATTCCA | aa320 (Ala to Ser) | Mutagenesis | pFastBac HTB - MDPV P1 | pFastBac HTB | 3.3 / Fig. 4 |
| A320S-R | GTAGTtgaTACTGGACCTGGTTGCAGTAGATACT |  |  |  |  |  |
| T321A-F | CAGTAGCTgctACACATACAGAAGATCAGGATTCCAG | aa321 (Thr to Ala) | Mutagenesis | pFastBac HTB - MDPV P1 | pFastBac HTB | 3.3 / Fig. 4 |
| T321A-R | ATGTGTagcAGCTACTGGACCTGGTTGCAGTA |  |  |  |  |  |
| Q327E-F | CAGAAGATgagGATTCCAGTATACCGGCTCAAAA | aa327 (Gln to Glu) | Mutagenesis | pFastBac HTB - MDPV P1 | pFastBac HTB | 3.3 / Fig. 4 |
| Q327E-R | GGAATCctcATCTTCTGTATGTGTAGTAGCTACTGGACC |  |  |  |  |  |
| I337L-F | GCTCAAAACACAttaGGAATTGCAAAAGACCCCTAC | aa337 (Ile to Leu) | Mutagenesis | pFastBac HTB - MDPV P1 | pFastBac HTB | 3.3 / Fig. 4 |
| I337L-R | CCtaaTGTGTTTTGAGCCGGTATACTGGAATC |  |  |  |  |  |
| I339L-F | GGAttaGCAAAAGACCCCTACAGGTCAGGCAG | aa339 (Ile to Leu) | Mutagenesis | pFastBac HTB - MDPV P1 | pFastBac HTB | 3.3 / Fig. 4 |
| I339L-R | GGGTCTTTTGCtaaTCCTATTGTGTTTTGAGCCGG |  |  |  |  |  |
| S349T-F | CAGTactCTGGCAGGAATTTCAGACATTATGG | aa349 (Ser to Thr) | Mutagenesis | pFastBac HTB - MDPV P1 | pFastBac HTB | 3.3 / Fig. 4 |
| S349T-R | TTCCTGCCAGagtACTGCCTGACCTGTAGGGGTC |  |  |  |  |  |
| I364V-F | GCAAGAAgtaGCACCAACTAATGGTGTAGGGTG | aa364 (Ile to Val) | Mutagenesis | pFastBac HTB - MDPV P1 | pFastBac HTB | 3.3 / Fig. 4 |
| I364V-R | TTGGTGCtacTTCTTGCTCATCTGGTACCATAATG |  |  |  |  |  |
| R373K-F | AGGGTGGaaaCCTTATGGATTGACCGTAACCAA | aa373 (Arg to Lys) | Mutagenesis | pFastBac HTB - MDPV P1 | pFastBac HTB | 3.3 / Fig. 4 |
| R373K-R | CATAAGGtttCCACCCTACACCATTAGTTGGTG |  |  |  |  |  |
| N391S-F | TCCTACAagtGCTGACCTACAAGTACTGGGAGC | aa391 (Asn to Ser) | Mutagenesis | pFastBac HTB - MDPV P1 | pFastBac HTB | 3.3 / Fig. 4 |
| N391S-R | GGTCAGCactTGTAGGAGCTGTTGTTGTGTTTTGT |  |  |  |  |  |
| A392S-F | CCTACAAATtcaGACCTACAAGTACTGGGAGCGC | aa392 (Ala to Ser) | Mutagenesis | pFastBac HTB - MDPV P1 | pFastBac HTB | 3.3 / Fig. 4 |
| A392S-R | AGGTCtgaATTTGTAGGAGCTGTTGTTGTGTTTT |  |  |  |  |  |
| Q395D-F | TGACCTAgatGTACTGGGAGCGCTACCTGGCA | aa395 (Gln to Asp) | Mutagenesis | pFastBac HTB - MDPV P1 | pFastBac HTB | 3.3 / Fig. 4 |
| Q395D-R | CCAGTACatcTAGGTCAGCATTTGTAGGAGCTGT |  |  |  |  |  |
| N301E-F | CTGGAGTgagGGAAACAAGGTTAATTTGAAGGACA | aa301 (Asn to Glu) | Mutagenesis | pFastBac HTB - GPV NP5 | pFastBac HTB | 3.4 / Fig. 5 |
| N301E-R | TGTTTCCctcACTCCAGATGTTCCAGTTTGCAT |  |  |  |  |  |
| N301R-F | TGGAGTcgcGGAAACAAGGTTAATTTGAAGGACA | aa301 (Asn to Arg) | Mutagenesis | pFastBac HTB - GPV NP5 | pFastBac HTB | 3.4 / Fig. 5 |
| N301R-R | TTGTTTCCgcgACTCCAGATGTTCCAGTTTGCAT |  |  |  |  |  |
| N301W-F | CTGGAGTtggGGAAACAAGGTTAATTTGAAGGACA | aa301 (Asn to Trp) | Mutagenesis | pFastBac HTB - GPV NP5 | pFastBac HTB | 3.4 / Fig. 5 |
| N301W-R | TGTTTCCccaACTCCAGATGTTCCAGTTTGCAT |  |  |  |  |  |
| N301G-F | TGGAGTggcGGAAACAAGGTTAATTTGAAGGACA | aa301 (Asn to Gly) | Mutagenesis | pFastBac HTB - GPV NP5 | pFastBac HTB | 3.4 / Fig. 5 |
| N301G-R | TTGTTTCCgccACTCCAGATGTTCCAGTTTGCAT |  |  |  |  |  |
| N301Q-F | CTGGAGTcagGGAAACAAGGTTAATTTGAAGGACA | aa301 (Asn to Gln) | Mutagenesis | pFastBac HTB - GPV NP5 | pFastBac HTB | 3.4 / Fig. 5 |
| N301Q-R | TGTTTCCctgACTCCAGATGTTCCAGTTTGCAT |  |  |  |  |  |
| N301A-F | TGGAGTgcgGGAAACAAGGTTAATTTGAAGGACA | aa301 (Asn to Ala) | Mutagenesis | pFastBac HTB - GPV NP5 | pFastBac HTB | 3.4 / Fig. 5 |
| N301A-R | TTGTTTCCcgcACTCCAGATGTTCCAGTTTGCAT |  |  |  |  |  |
| K301E-F | CTGGAGTgagGGAAACAAAGTTTTTCTTAAAGACAGAG | aa301 (Lys to Glu) | Mutagenesis | pFastBac HTB - MDPV P1 | pFastBac HTB | 3.4 / Fig. 5 |
| K301E-R | TGTTTCCctcACTCCAGATTGACCAGTTCGCA |  |  |  |  |  |
| K301R-F | TGGAGTcgcGGAAACAAAGTTTTTCTTAAAGACAGAG | aa301 (Lys to Arg) | Mutagenesis | pFastBac HTB - MDPV P1 | pFastBac HTB | 3.4 / Fig. 5 |
| K301R-R | TTGTTTCCgcgACTCCAGATTGACCAGTTCGCA |  |  |  |  |  |
| K301W-F | CTGGAGTtggGGAAACAAAGTTTTTCTTAAAGACAGAG | aa301 (Lys to Trp) | Mutagenesis | pFastBac HTB - MDPV P1 | pFastBac HTB | 3.4 / Fig. 5 |
| K301W-R | TGTTTCCccaACTCCAGATTGACCAGTTCGCA |  |  |  |  |  |
| K301G-F | TGGAGTggcGGAAACAAAGTTTTTCTTAAAGACAGAG | aa301 (Lys to Gly) | Mutagenesis | pFastBac HTB - MDPV P1 | pFastBac HTB | 3.4 / Fig. 5 |
| K301G-R | TTGTTTCCgccACTCCAGATTGACCAGTTCGCA |  |  |  |  |  |
| K301Q-F | CTGGAGTcagGGAAACAAAGTTTTTCTTAAAGACAGAG | aa301 (Lys to Gln) | Mutagenesis | pFastBac HTB - MDPV P1 | pFastBac HTB | 3.4 / Fig. 5 |
| K301Q-R | TGTTTCCctgACTCCAGATTGACCAGTTCGCA |  |  |  |  |  |
| K301A-F | CTGGAGTgcaGGAAACAAAGTTTTTCTTAAAGACAGAG | aa301 (Lys to Ala) | Mutagenesis | pFastBac HTB - MDPV P1 | pFastBac HTB | 3.4 / Fig. 5 |
| K301A-R | TGTTTCCtgcACTCCAGATTGACCAGTTCGCA |  |  |  |  |  |
| T62A-F | AGTGGAgcgTCTCAAGATGCAAATGTCCAGTATG | aa62 (Thr to Ala) | Mutagenesis | pFastBac HTB - GPV NP5 | pFastBac HTB | 3.7 / Fig. 9 |
| T62A-R | TCTTGAGAcgcTCCACTGGTAATCGCTTTGTAGAT |  |  |  |  |  |
| S63A-F | AACCgcgCAAGATGCAAATGTCCAGTATGCAG | aa63 (Ser to Ala) | Mutagenesis | pFastBac HTB - GPV NP5 | pFastBac HTB | 3.7 / Fig. 9 |
| S63A-R | TTGCATCTTGcgcGGTTCCACTGGTAATCGCTTTG |  |  |  |  |  |
| Q64A-F | AACCTCTgcgGATGCAAATGTCCAGTATGCAGG | aa64 (Gln to Ala) | Mutagenesis | pFastBac HTB - GPV NP5 | pFastBac HTB | 3.7 / Fig. 9 |
| Q64A-R | TTGCATCcgcAGAGGTTCCACTGGTAATCGCTT |  |  |  |  |  |
| D65A-F | CTCTCAAgctGCAAATGTCCAGTATGCAGGATAC | aa65 (Asp to Ala) | Mutagenesis | pFastBac HTB - GPV NP5 | pFastBac HTB | 3.7 / Fig. 9 |
| D65A-R | CATTTGCagcTTGAGAGGTTCCACTGGTAATCG |  |  |  |  |  |
| Q182A-F | CACCAACgcaAATGGAGCACGGTTCAATGACC | aa182 (Gln to Ala) | Mutagenesis | pFastBac HTB - GPV NP5 | pFastBac HTB | 3.7 / Fig. 9 |
| Q182A-R | CTCCATTtgcGTTGGTGTGCATGGTGCAGTAC |  |  |  |  |  |
| Y293A-F | CAGATAATgcgGCAAACTGGAACATCTGGAGTAATG | aa293 (Tyr to Ala) | Mutagenesis | pFastBac HTB - GPV NP5 | pFastBac HTB | 3.7 / Fig. 9 |
| Y293A-R | GTTTGCcgcATTATCTGTTCCGCCTGGATAGG |  |  |  |  |  |
| N295A-F | TTATGCAgcgTGGAACATCTGGAGTAATGGAAAC | aa295 (Asn to Ala) | Mutagenesis | pFastBac HTB - GPV NP5 | pFastBac HTB | 3.7 / Fig. 9 |
| N295A-R | TGTTCCAcgcTGCATAATTATCTGTTCCGCCTG |  |  |  |  |  |
| W296A-F | TGCAAACgcgAACATCTGGAGTAATGGAAACAAGG | aa296 (Trp to Ala) | Mutagenesis | pFastBac HTB - GPV NP5 | pFastBac HTB | 3.7 / Fig. 9 |
| W296A-R | AGATGTTcgcGTTTGCATAATTATCTGTTCCGCC |  |  |  |  |  |
| N297A-F | AAACTGGgcgATCTGGAGTAATGGAAACAAGGTTAA | aa297 (Asn to Ala) | Mutagenesis | pFastBac HTB - GPV NP5 | pFastBac HTB | 3.7 / Fig. 9 |
| N297A-R | TCCAGATcgcCCAGTTTGCATAATTATCTGTTCCG |  |  |  |  |  |
| W299A-F | GAACATCgcgAGTAATGGAAACAAGGTTAATTTGAAGG | aa299 (Trp to Ala) | Mutagenesis | pFastBac HTB - GPV NP5 | pFastBac HTB | 3.7 / Fig. 9 |
| W299A-R | CATTACTcgcGATGTTCCAGTTTGCATAATTATCTGT |  |  |  |  |  |
| S300A-F | CATCTGGgcgAATGGAAACAAGGTTAATTTGAAGG | aa300 (Ser to Ala) | Mutagenesis | pFastBac HTB - GPV NP5 | pFastBac HTB | 3.7 / Fig. 9 |
| S300A-R | TTCCATTcgcCCAGATGTTCCAGTTTGCATAATT |  |  |  |  |  |
| T350A-F | CACTgcgGCAGGAATAAGTGATGTTATGGTCA | aa350 (Thr to Ala) | Mutagenesis | pFastBac HTB - GPV NP5 | pFastBac HTB | 3.7 / Fig. 9 |
| T350A-R | TTATTCCTGCcgcAGTGCTGCCAGATCTGTATGGAT |  |  |  |  |  |
